# T Cell Activating Thermostable Self‐Assembly Nanoscaffold Tailored for Cellular Immunity Antigen Delivery

**DOI:** 10.1002/advs.202303049

**Published:** 2023-07-03

**Authors:** Jinsong Zhang, Jianghua Yang, Qianlin Li, Ruihao Peng, Shoudong Fan, Huaimin Yi, Yuying Lu, Yuanli Peng, Haozhen Yan, Lidan Sun, Jiahai Lu, Zeliang Chen

**Affiliations:** ^1^ One Health Center of Excellence for Research and Training School of Public Health Sun Yat‐sen University Guangzhou 510080 China; ^2^ NMPA Key Laboratory for Quality Monitoring and Evaluation of Vaccines and Biological Products Guangzhou 510080 China; ^3^ Key Laboratory of Tropical Diseases Control Sun Yat‐sen University Ministry of Education Guangzhou 510080 China; ^4^ Key Laboratory of Livestock Infectious Diseases Ministry of Education Shenyang Agricultural University Shenyang 110866 China; ^5^ Liaoning Technology Innovation Center of Nanomaterials for Antibiotics Reduction and Replacement Fengcheng 118199 China; ^6^ Research Institute of Sun Yat‐sen University in Shenzhen Shenzhen 518057 China; ^7^ Hainan Key Novel Thinktank “Hainan Medical University ‘One Health’ Research Center” Haikou 571199 China; ^8^ Key Laboratory of Zoonose Prevention and Control at Universities of Inner Mongolia Autonomous Region Medical College Inner Mongolia Minzu University Tongliao 028000 China

**Keywords:** antigen delivery, protein engineering, self‐assembly, T cell‐mediated immunity, thermostable nanoscaffold

## Abstract

Antigen delivery based on non‐virus‐like particle self‐associating protein nanoscffolds, such as *Aquifex aeolicus* lumazine synthase (AaLS), is limited due to the immunotoxicity and/or premature clearance of antigen‐scaffold complex resulted from triggering unregulated innate immune responses. Here, using rational immunoinformatics prediction and computational modeling, we screen the T epitope peptides from thermophilic nanoproteins with the same spatial structure as hyperthermophilic icosahedral AaLS, and reassemble them into a novel thermostable self‐assembling nanoscaffold RP_T_ that can specifically activate T cell‐mediated immunity. Tumor model antigen ovalbumin T epitopes and the severe acute respiratory syndrome coronavirus 2 receptor‐binding domain are loaded onto the scaffold surface through the SpyCather/SpyTag system to construct nanovaccines. Compared to AaLS, RP_T_‐constructed nanovaccines elicit more potent cytotoxic T cell and CD4^+^ T helper 1 (Th1)‐biased immune responses, and generate less anti‐scaffold antibody. Moreover, RP_T_ significantly upregulate the expression of transcription factors and cytokines related to the differentiation of type‐1 conventional dendritic cells, promoting the cross‐presentation of antigens to CD8^+^ T cells and Th1 polarization of CD4^+^ T cells. RP_T_ confers antigens with increased stability against heating, freeze‐thawing, and lyophilization with almost no antigenicity loss. This novel nanoscaffold offers a simple, safe, and robust strategy for boosting T‐cell immunity‐dependent vaccine development.

## Graphical abstract

The study redirects antigenic properties of the thermostable self‐assembly nanoscaffold *Aquifex aeolicus* lumazine synthase (AaLS) for T‐cell immunity‐dependent antigen delivery. Compared to AaLS, the ab initio designed nanoscaffold‐constructed nanovaccines elicit more potent cytotoxic T cell and CD4^+^ T helper 1‐biased immune responses, and generate less anti‐scaffold antibody. The novel nanoscaffold offers a robust strategy for boosting T‐cell immunity‐based vaccine development.

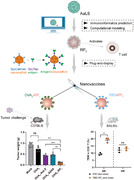

## 1 Introduction

Antigen delivery via nanoscale scaffolds is an attractive strategy in vaccine development.^[^ ^1^ ^]^ Nanoscaffolds have a well‐defined spatial structure that allows the introduction of multiple copies of antigens and are highly efficient in tissue targeting, circulation prolonging, and preferential uptake with professional antigen‐presenting cells (APCs).^[^ ^2^, ^3^ ^]^ Nanoproteins have been particularly attractive as multimeric antigen delivery platforms in recent years due to their atomically precise assembly over non‐biosynthetic polymers.^[^ ^4^ ^]^ These proteins, which are derived from biological expression systems, display antigens externally and/or internally in a spatially controlled manner with favorable biocompatibility and modifiability.^[^ ^5^, ^6^ ^]^

Protein nanoscaffold‐based antigen presentation platforms include virus‐like particles (VLPs) and non‐VLP self‐associating proteins.^[^ ^4^ ^]^ VLPs consist of a self‐assembled viral envelope or capsid proteins, and have been extensively used as a platform in clinical trials and commercially approved vaccines. For instance, hepatitis B virus and human papillomavirus vaccines based on the VLPs of hepatitis B virus and human papillomavirus, respectively, have been approved for production.^[^ ^7^ ^]^ However, poor stability, low expression levels, and host system contamination limit the use of VLPs as a universal platform.^[^ ^8^, ^9^ ^]^ In contrast to VLPs, non‐VLP self‐associating proteins are highly oligomeric structures assembled with single protein components that are rapidly derived from biological expression systems, which have higher stability, safety, and universality as vaccine carriers, including the ferritin,^[^ ^10^, ^11^ ^]^ *Aquifex aeolicus* lumazine synthase (AaLS),^[^ ^12^, ^13^ ^]^ dihydrolipoyl acetyltransferase,^[^ ^14^ ^]^ and heat shock proteins.^[^ ^15^ ^]^ Various ferritin‐based vaccines are being tested in clinical trials, including two influenza trials that have recently completed phase I (NCT03186781 and NCT03814720), and ongoing trials for Epstein‐Barr virus (NCT04645147), severe acute respiratory syndrome coronavirus 2 (SARS‐CoV‐2) (NCT04784767), and influenza (NCT04579250). An AaLS‐based human immunodeficiency virus vaccine, eOD‐GT8, has recently completed a phase I human clinical trial (NCT03547245).

The protein nanoscaffold‐based antigen delivery platform is clinically successful. It can enhance the efficiency of antigen delivery to the immune system and act as an adjuvant to stimulate innate immunity. For instance, some VLP epitopes serve as co‐stimulatory molecules and are processed through the endolysosomal pathways, which allows antigen cross‐presentation, boosting humoral and cellular immunity.^[^ ^16^ ^]^ The geometry and multivalency of VLP or non‐VLP self‐associating proteins present pathogen‐associated molecular patterns that trigger innate immune recognition through pattern recognition receptors, most commonly toll‐like receptors (TLRs).^[^ ^17^, ^18^ ^]^ However, this unregulated innate immune stimulation of nanoparticles may result in immunotoxicity and/or premature clearance of the antigen‐scaffold complex. One possible way for activating a given immune pathway is to redirect the intrinsic immunogenicity of the scaffold. However, few studies have shown that specific immune types can be activated by tailoring the antigenic properties of nanoparticles.

In this study, using rational immunoinformatics prediction and computational modeling, we screened the T epitope peptides from thermophilic nanoproteins with the same spatial structure as hyperthermophilic icosahedral AaLS, and reassemble them into a novel nanoscaffold that can specifically activate T cell‐mediated immunity. The immune efficacy of the nanoscaffold antigen delivery system was assessed in tumor and severe acute respiratory syndrome coronavirus 2 (SARS‐CoV‐2) models, and the downstream molecular mechanism of the nanoscaffold triggering an T cell‐based immune response was further investigated. We also fully evaluated the safety, stability, and antigen‐cross presentation of the antigen‐nanoscaffold complex. This novel nanoscaffold simultaneously triggered T cell‐based immunity and enhanced antigen stability, potentially accelerating T‐cell immunity‐dependent vaccines development.

## 2 Results

### 2.1 Ab Initio Design of Thermostable T Cell‐Activating Self‐Assembling Nanoscaffolds

**Figure 1:**
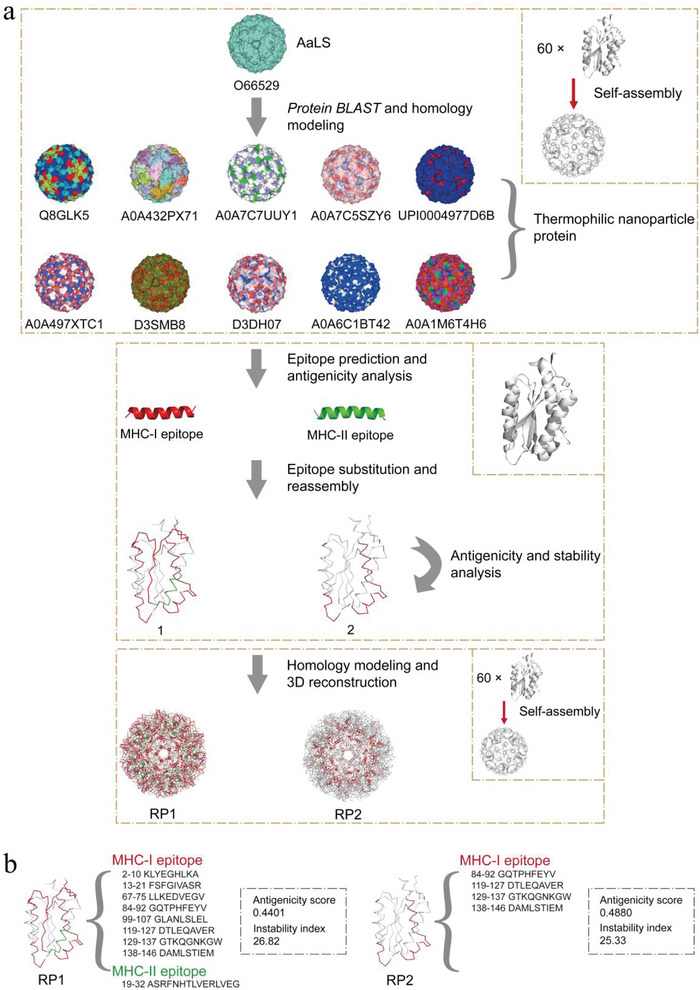
Ab initio design of T cell‐activating thermostable nanoscaffolds. a) Ab initio design flowchart of T cell‐activating thermostable nanoscaffolds. RP1 and RP2 could self‐assemble icosahedral structures with 60 identical subunits. b) The subunit structure and introduced epitope types of RP1 and RP2. The antigenicity score and instability index of RP1 and RP2 are shown in the dashed box. An antigenicity score greater than 0.4000 classifies the protein as “Probable ANTIGEN” (http://www.ddg‐pharmfac.net/vaxijen/VaxiJen/VaxiJen.html). An instability index less than 40.00 classifies the protein as stable (https://web.expasy.org/protparam/).

The primary amino acid sequence of the AaLS subunit was retrieved from the UniProt Knowledgebase with accession number O66529. Protein BLAST was performed to search for homologous sequences from thermophiles with a spatial structure similar to AaLS. More than a dozen native homologous sequences were matched. Surprisingly, the experimental 3D structures of these sequences were not available in the Protein Data Bank. Based on the known X‐ray crystal structure of AaLS, 3D structures of homologous sequences were generated through homology modeling. Ten sequences with the same 3D structure as AaLS were screened, as shown in **Figure** **1**a and Figure S1, Supporting Information. All homologous sequences self‐assembled into an icosahedral structure with 60 identical subunits.

We first analyzed the antigenicity of these homologous sequences. Surprisingly, only AaLS showed “Probable ANTIGEN.” In contrast, all ten homologous sequences showed “Probable NON‐ANTIGEN,” thus, they cannot form potential antigens. Different functionalized T‐dominant epitope peptides with strong antigenicity were predicted from the homologous sequences and screened according to their T cell recognition ability. Ten MHC class I (MHCI) molecule‐binding epitopes and seven MHC class II (MHCII) molecule‐binding epitopes were found. The T‐dominant epitopes substituted the sequences at the same position of the AaLS subunit to obtain reassembled protein (RP) sequences, and some amino acids were mutated appropriately to maintain the protein structural stability. The antigenicity and stability of the RP sequences were further analyzed, and 3D structures of the RP sequences were generated via homology modeling. By excluding RPs with poor spatial structure quality, we obtained two novel T cell‐activating icosahedral RP1 and RP2 proteins (Figure 1a,b; Figure S1, Supporting Information). Herein, B cell epitopes were not incorporated to minimize anti‐scaffold antibody responses.

### 2.2 Construction of a Plug‐and‐Display Antigen Delivery Strategy Using the Designed Nanoscaffolds

**Figure 2:**
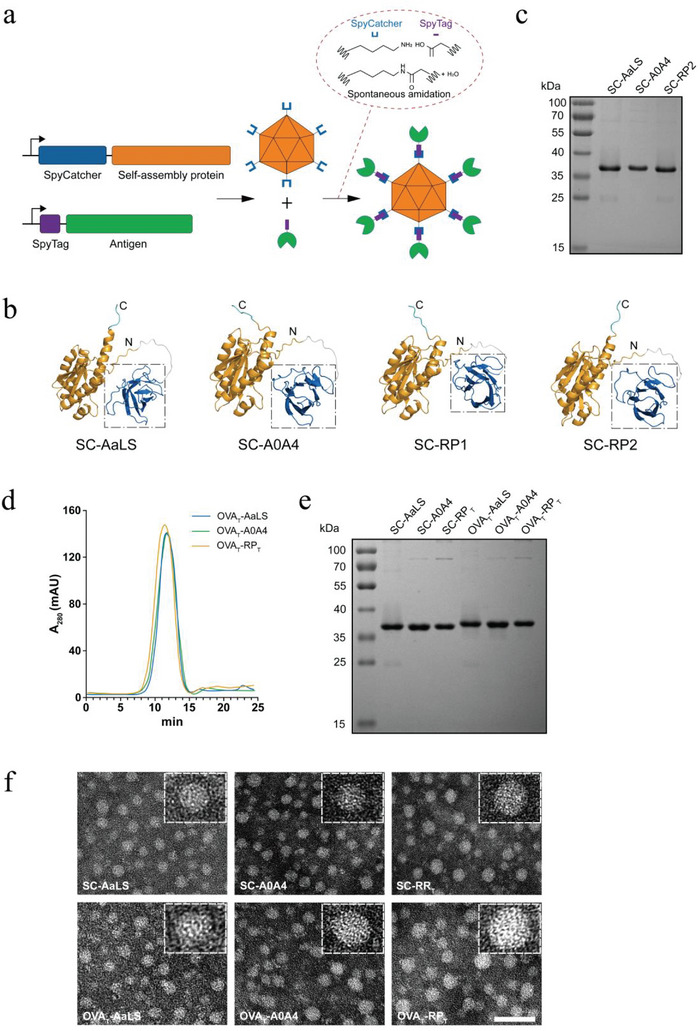
Construction and characterization of a plug‐and‐display antigen delivery system. a) Plug‐and‐display schematic diagram. b) Alpha Fold 2 prediction of scaffold monomer fusion structure. The SC (blue) in the dashed box is exposed near the monomer's N‐terminus via a flexible linker (white), with no effect on scaffold monomer folding (yellow). The bulging C‐terminus is the 6 × His tag (cyan). c) The purified SC‐AaLS, SC‐A0A4, and SC‐RP2 were analyzed using SDS‐PAGE with Coomassie staining. The nanoscaffolds were solubly expressed in *E. coli* and were purified from the supernatant of the cell lysate using immobilized metal ion affinity chromatography and SEC. d) SEC of OVA_T_‐AaLS, OVA_T_‐A0A4, and OVA_T_‐RP_T_ at A_280_. e) Coomassie staining of SC‐AaLS, SC‐A0A4, SC‐RP_T_, OVA_T_‐AaLS, OVA_T_‐A0A4, and OVA_T_‐RP_T_. f) Negatively stained transmission electron microscopy images of SC‐AaLS, SC‐A0A4, SC‐RP_T_, OVA_T_‐AaLS, OVA_T_‐A0A4, and OVA_T_‐RP_T_. Nanoparticles with equal magnification are shown in the dashed box. Scale bar: 50 nm.

Poor yields and the formation of inclusion bodies in bacterial expression systems are common concerns with the fusion expression of scaffolds and antigens, severely restricting the utilization of scaffold delivery systems. A plug‐and‐display antigen delivery strategy that emerged in recent years has been broadly used in delivering nanovaccines.^[^ ^19^, ^20^, ^21^, ^22^ ^]^ This method utilizes the spontaneous isopeptide bond formed between the protein domain SpyCatcher (SC) (114 aa) and its peptide partner SpyTag (ST) (16 aa) to directionally load the antigen onto the nanoparticle surface (**Figure** **2**a). A modular assembly of separate antigens and scaffolds may offer optimized production conditions for both components and shorten the cycle from the design to production of new vaccines.

The X‐ray crystal structure of AaLS has shown that both the N‐ and C‐terminals of the monomers are surface‐exposed, allowing the insertion of foreign antigenic sequences at these two sites.^[^ ^23^ ^]^ Computational modeling predicted that the subunit termini of RP1 and RP2 were also exposed on the particle surface. Therefore, SC was genetically fused at the N‐terminus of RPs with a flexible linker (GGSGGSGGSGGS) to create the plug‐and‐display nanoscaffolds, also adding a 6 × His tag at the C‐terminus to enhance downstream purification. AaLS and the non‐antigenic natural homologous protein A0A497XTC1 (A0A4) were used for subsequent comparisons. A0A4 is derived from *Hydrogenivirga caldilitoris*, an extremely thermophilic, hydrogen‐ and sulfur‐oxidizing bacterium isolated from a coastal hydrothermal field.^[^ ^24^ ^]^ We further predicted the monomer fusion structure of these scaffolds using Alpha Fold 2. The prediction model clearly showed that the SC was exposed at the right position near the N‐terminus of the monomer via a flexible linker, and had no effect on protein folding (Figure 2b).

Four constructs, SC‐AaLS, SC‐A0A4, SC‐RP1, and SC‐RP2, were expressed in *Escherichia coli* (*E. coli*). All constructs were expressed in soluble form, except SC‐RP1, and SC‐RP2 had the highest purification yield of up to 34.82 mg L^−1^ (Figure 2c; Figure S1a,b, Supporting Information). The N‐terminus of tumor model antigen ovalbumin T epitopes (OVA_T_) was genetically fused with ST (RGVPHIVMVDAYKRYK) to covalently conjugate the SC‐modified scaffolds. Purified SC‐AaLS, SC‐A0A4, and SC‐RP2 were incubated with ST‐OVA_T_ to generate OVA_T_‐AaLS, OVA_T_‐A0A4, and OVA_T_‐RP_T_ (RP2 was represented by RP_T_) nanovaccines in conventional buffer without any enzyme. Theoretically, the nanoscaffold surface allows the presentation of 60 copies of the OVA_T_ antigen peptide. The antigen‐conjugated nanoscaffolds were purified via size exclusion chromatography (SEC) and analyzed using sodium dodecyl sulphate‐polyacrylamide gel electrophoresis (SDS‐PAGE), which showed a monodisperse SEC profile and nearly 100% conjugation (Figure 2d,e). Transmission electron microscopy revealed homogeneous spherical nanoparticles (Figure 2f). The particle sizes of SC‐AaLS and SC‐A0A4 were ≈21–23 nm, whereas the peptide reassembly generated SC‐RP_T_, and had an expanded spherical structure of ≈22–25 nm. The particle sizes of the three nanoscaffolds increased by ≈1–2 nm after antigen conjugation.

### 2.3 The RP_T_ Antigen Delivery System Is Extremely Robust

**Figure 3:**
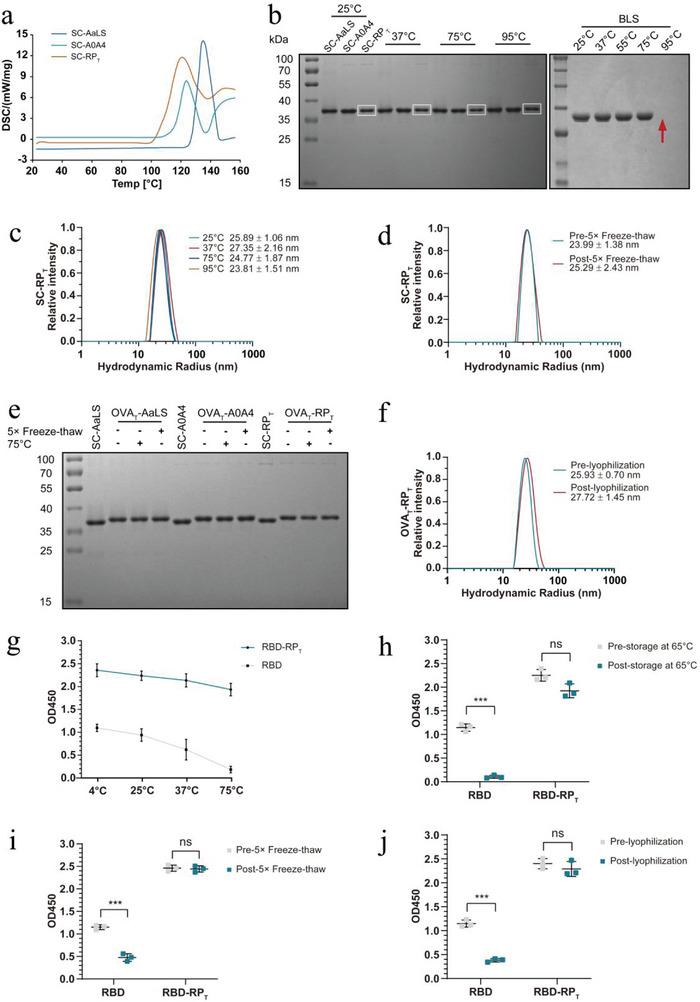
Stability analysis of the RP_T_ antigen delivery system. a) The calorimetric melting curves of SC‐AaLS, SC‐A0A4, and SC‐RP_T_ were measured using DSC. b) The solubility of SC‐AaLS, SC‐A0A4, and SC‐RP_T_ after 2 h of incubation at different temperatures was determined using SDS‐PAGE with Coomassie staining. SC‐RP_T_ is in the white square. The red arrow indicates the degraded BLS. c) The integrity of SC‐RP_T_ after 2 h of incubation at different temperatures was determined using DLS (*n* = 3). d) The integrity of SC‐RP_T_ after 5 × freeze‐thawing was determined using DLS (*n* = 3). e) SC‐AaLS, SC‐A0A4, and SC‐RP_T_ retained their reactivity after heating at 75 °C or 5 × freeze‐thawing. The reactions of SC‐AaLS, SC‐A0A4, and SC‐RP_T_ with ST‐OVA_T_ at 4 °C for 16 h were analyzed using SDS‐PAGE with Coomassie staining. f) The integrity of OVA_T_‐RP_T_ after lyophilization was determined using DLS (*n* = 3). g) The immunoreactivity of RBD‐RP_T_ and free RBD after 2 h of incubation at different temperatures was determined using ELISA. h) The immunoreactivity of RBD‐RP_T_ and free RBD after storage for 2 weeks at 65 °C was determined using ELISA. i) The immunoreactivity of RBD‐RP_T_ and free RBD after 5 × freeze‐thawing and j) lyophilization was determined using ELISA. Data are expressed as the mean ± SEM. *p < 0.05, **p < 0.01, ***p < 0.001; ns represents not significant.

To transition from small‐scale laboratory manufacturing to clinical and field settings, nanoscaffold platforms should be robust and tolerant to long‐term storage and varying temperatures to minimize antigen loss.^[^ ^25^ ^]^ Differential scanning calorimetry (DSC) showed that SC‐RP_T_ had a similar apparent melting temperature of 119.1 °C with SC‐AaLS and SC‐A0A4, which are 135.7 °C and 124.5 °C, respectively (**Figure** **3**a). At 95 °C, the non‐thermophilic *Brucella* lumazine synthase(BLS) was almost completely degraded, while SC‐RP_T_ maintained a high solubility range of 83–89% (Figure 3b). Based on dynamic light scattering (DLS) data, SC‐RP_T_ also remained well‐formed after heating, and its hydrodynamic radius fluctuated slightly with increasing temperature (Figure 3c; Figure S3a, Supporting Information). SC‐RP_T_ could tolerate storage at 37 °C and 65 °C for up to one month and maintained a soluble protein ratio of more than 80% (Figure S3b, Supporting Information). Freezing and dehydration stresses often inactivate proteins by unfolding or altering their structures.^[^ ^26^, ^27^ ^]^ After five rounds of freeze‐thawing, the soluble protein fraction of SC‐RP_T_ did not change, and the particles remained well‐formed (Figure 3d; Figure S3c,d, Supporting Information). The conjunction efficiency of SC‐RP_T_ with ST‐OVA_T_ was unaffected after heating and cyclic freeze‐thawing, which indicated that SC‐RP_T_ retained good reactivity (Figure 3e). SC‐RP_T_ was further exposed to a wide range of pH values (0.1, 4, 7, and 14) and salt concentrations (0, 50, 100, and 500 mm). SC‐RP_T_ was found to aggregate under strong acid‐base and high salt concentrations. After restoring the original buffered condition, the nanoscaffold was reassembled (Figure S3e, Supporting Information). The robust SC‐RP_T_ gave the antigen high stability during heating and freeze‐thawing (Figure S3f–i, Supporting Information). At least 77% of the protein remained in the soluble fraction up to 75 °C, and no degradation was observed (Figure S3g, Supporting Information). We also assessed stability during lyophilization. OVA_T_‐RP_T_ could be lyophilized and reconstituted without damaging the particle shape (Figure 3f).

We demonstrated the extreme robustness of SC‐RP_T_ and its ability to maintain the integrity of the surface antigens under harsh storage conditions. More importantly, the original antigenicity of the antigen after different treatments should be retained. We further analyzed whether SARS‐CoV‐2 receptor‐binding domain (RBD)‐conjugated nanoscaffolds lost their antigenicity in extreme environments using an enzyme‐linked immunosorbent assay (ELISA) against SARS‐CoV‐2 spike protein RBD mAb. Similarly, the N‐terminus of the RBD was genetically fused to ST to covalently conjugate SC‐RP_T_ and generate RBD‐RP_T_. Compared to free RBD, RBD‐RP_T_ conjugated with equal moles of RBD retained initial antibody recognition after heating at 75 °C, cyclic freeze–thawing, and lyophilization, with no apparent antigenicity loss (Figure 3g,i,j). When stored at 65 °C for 2 weeks, the antigen reactivity of RBD‐RP_T_ did not change markedly, whereas that of the free RBD was almost exclusively lost (Figure 3h). We also observed that the antibody‐binding capacity of RBD‐RP_T_ was dramatically higher than that of free RBD pre‐ and post‐treatment.

### 2.4 RP_T_‐Derived Nanovaccines Induce Potent Antigen Presentation and T Cell Immune Response

**Figure 4:**
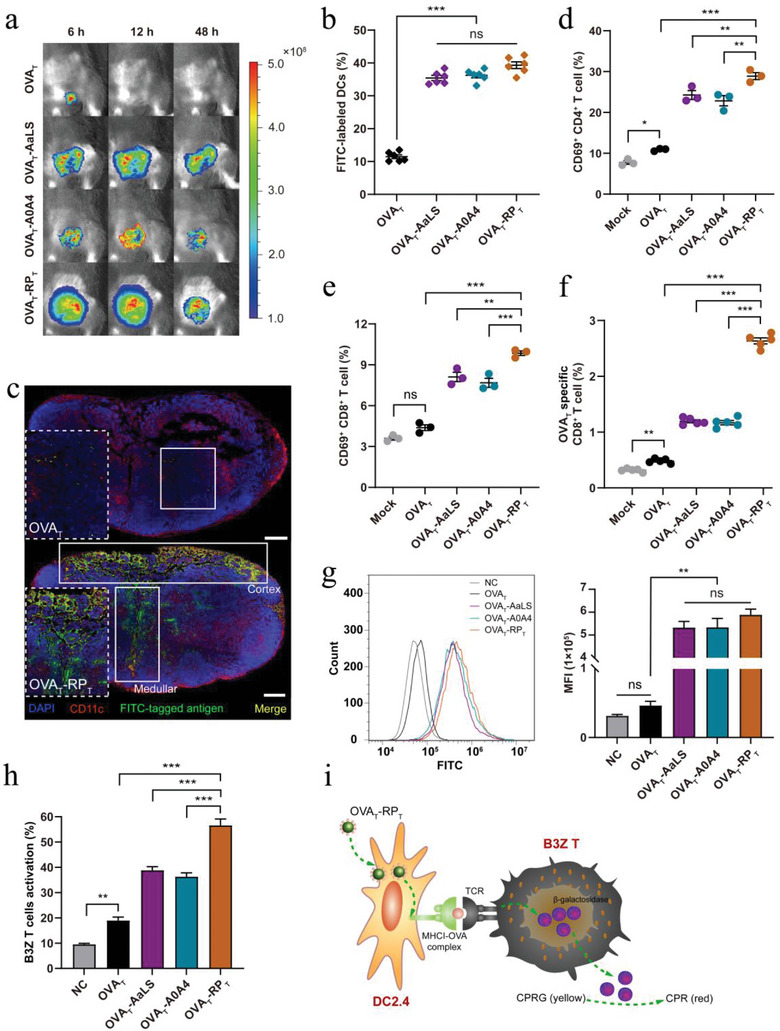
Antigen presentation and T cell immune response of RP_T_‐derived tumor nanovaccines. a) Antigen depot at the inguinal region of mice. b) Quantification of the percentage of FITC‐positive DCs in the inguinal lymph nodes of immunized mice at 4 h post‐administration (*n* = 6). c) Immunofluorescence co‐localization images of the inguinal lymph nodes of immunized mice at 4 h post‐administration. Cryosections of inguinal lymph nodes were prepared, and DCs were fluorescently labeled with antibodies against CD11c (red). Green staining indicates FITC‐tagged antigens. Blue staining indicates DAPI‐stained nuclei. Scale bar: 200 µm. d) Quantification of the percentage of CD69^+^ CD4^+^ and e) CD69^+^ CD8^+^ T cells in the spleen of immunized mice at 7 days post‐administration (*n* = 3). f) Quantification of OVA_T_‐specific CD8^+^ T cells in spleens (*n* = 5), as determined with staining using an APC‐conjugated SIINFEKL‐H‐2K^b^ tetramer. g) Representative flow cytometry plots and quantification of the mean fluorescence intensity (MFI) in DC2.4 cells (*n* = 3). NC: negative control. h) Quantification of B3Z T cell activation (*n* = 3) and i) B3Z antigen cross‐presentation system schematic diagram. DC2.4 cells were treated with free OVA_T_ peptides or the nanovaccines and co‐cultured with B3Z T cells that produce *β*‐galactosidase in response to antigen cross‐presentation. Data are expressed as the mean ± SEM. *p < 0.05, **p < 0.01, ***p < 0.001; ns represents not significant.

A preeminent antigen delivery system can efficiently drain and accumulate in the lymph nodes, which is easier to capture by APCs to enhance antigen presentation and T cell activation.^[^ ^4^, ^5^, ^28^ ^]^ We conducted antigen presentation tests to elucidate how the RP_T_‐derived nanovaccines are recognized and processed by the host immune system in vivo and in vitro. C57BL/6 mice were immunized with equimolar amounts of fluorescein isothiocyanate (FITC)‐labeled OVA_T_, OVA_T_‐AaLS, OVA_T_‐A0A4, and OVA_T_‐RP_T_ in the inguinal region, and antigen deposition at the injection sites was evaluated. As shown in **Figure** **4**a, the three nanovaccines, especially OVA_T_‐RP_T_, could efficiently accumulate in the mouse lymph nodes, and the fluorescence intensity decreased slowly over time post‐injection. Free OVA_T_ did not show any lymph node retention at 12 h post‐injection. It may be that free OVA_T_ peptides with extremely small molecular weights can be quickly cleared in the body, making it hardly accumulate in large quantities in lymph nodes. We continuously quantitatively observed the retention of free OVA_T_, OVA_T_‐AaLS, OVA_T_‐A0A4, and OVA_T_‐RP_T_ in inguinal lymph nodes through flow cytometry. At 96 h post‐injection, OVA_T_‐AaLS and OVA_T_‐A0A4 were almost completely metabolized, while OVA_T_‐RP_T_ still maintained partial fluorescence intensity until it completely disappeared at 120 h (Figure S4a, Supporting Information). At 4 h post‐injection, the inguinal lymph nodes were isolated, and dendritic cells (DCs) were collected to detect the percentage of FITC‐tagged DCs. The results showed that the percentage of fluorescently labeled DCs induced by OVA_T_‐AaLS, OVA_T_‐A0A4, and OVA_T_‐RP_T_ was significantly higher than that of free OVA_T_ (Figure 4b; Figure S4b, Supporting Information). Owing to its expanded spherical structure, OVA_T_‐RP_T_ seemed to be preferentially captured by APCs compared to OVA_T_‐AaLS and OVA_T_‐A0A4, although there was no significant statistical difference. Immunostaining of lymph nodes also showed that FITC‐labeled OVA_T_‐RP_T_ was markedly colocalized with DCs in the cortical and medullary regions, with a significantly higher fluorescence intensity than OVA_T_ (Figure 4c).

C57BL/6 mice were euthanized seven days post‐vaccination, and the activation of different T cells within the spleen was assessed. The nanovaccines elicited a significant increase in the proportion of activated CD8^+^ (CD3^+^ CD8^+^ CD69^+^) and CD4^+^ (CD3^+^ CD4^+^ CD69^+^) T cells in contrast to free OVA_T_, with the highest proportion in the OVA_T_‐RP_T_ group (Figure 4d,e; Figure S4c, Supporting Information). More importantly, the OVA_T_‐RP_T_ induced more OVA_T_‐specific CD8^+^ T cells (Figure 4f; Figure S4d, Supporting Information) that secreted more IFN‐*γ* upon stimulation with OVA_T_ peptides in vitro (Figure S4e, Supporting Information). With respect to the cancer milieu, treatments that ensure a shift toward the anti‐tumor CD4^+^ T helper 1 (Th1) response are essential, while maintaining a low T helper 2 (Th2) response is critical to ensure a tumor‐specific immune response.^[^ ^29^ ^]^ Therefore, we detected different CD4^+^ T cell populations in the spleens of the vaccinated mice. At seven days post‐prime vaccination, OVA_T_‐RP_T_ elicited Th1‐biased IFN‐*γ* ^+^ CD4^+^ T cells than other controls, whereas the percentages of Th2‐skewed IL‐4^+^ CD4^+^ T cells did not differ across the different groups (Figure S5a,b, Supporting Information). Additionally, OVA_T_‐RP_T_‐immunized mice produced higher levels of Th1 cytokines IL‐2, IL‐12p70, IFN‐*γ*, and TNF‐a in their peripheral blood than other controls, whereas the levels of Th2 cytokines IL‐4 did not differ across the different groups (Figure S5c, Supporting Information).

DC2.4 cells were co‐incubated with FITC‐labeled OVA_T_, OVA_T_‐AaLS, OVA_T_‐A0A4, and OVA_T_‐RP_T_. The uptake of nanovaccines boosted the internalization of the antigens by 1200% compared to free OVA_T_ (Figure 4g; Figure S4f, Supporting Information). Similarly, although there was no statistically significant difference, OVA_T_‐RP_T_ was preferentially captured by DCs compared to OVA_T_‐AaLS and OVA_T_‐A0A4. Compared to OVA_T_‐AaLS and OVA_T_‐A0A4, OVA_T_‐RP_T_ significantly promoted the expression and secretion of mature DCs CD40, CD80, CD86, IFN‐*α*, and IL‐12p70, which are crucial for inducing DCs to cross‐present antigens to T cells (Figure S6a–e, Supporting Information). Given that the OVA_T_ peptide is a canonical CD8^+^ T cell epitope, we evaluated the cross‐presentation of OVA_T_‐RP_T_ to CD8^+^ T cells via the MHCI molecular pathway using the B3Z antigen presentation system (Figure 4i). The system utilizes DC2.4 to capture exogenous antigen OVA_T_‐RP_T_, which enters cells and forms MHCI‐OVA_T_ complexes with MHCI molecules, and presents the complexes to the cell surface. B3Z T cells, CD8^+^ T cell hybridomas, express a T cell receptor (TCR) that specifically recognizes MHCI‐OVA_T_ (SIINFEKL) in the context of H‐2K^b^ and produces *β*‐galactosidase in response to MHCI antigen cross‐presentation. DC2.4 cells treated with OVA_T_‐RP_T_ induced more *β*‐galactosidase in B3Z T cells than those treated with OVA_T_‐AaLS and OVA_T_‐A0A4, showing a marked improvement in antigen cross‐presentation (Figure 4h). In conclusion, OVA_T_‐RP_T_ could be efficiently processed by APC and cross‐presented to specific CD8^+^ T cells, which is critical for inducing antitumor immunity and direct tumor killing.

### 2.5 RP_T_‐Derived Tumor Nanovaccines Show Excellent Antitumor Immunity and Direct Tumor Killing

**Figure 5:**
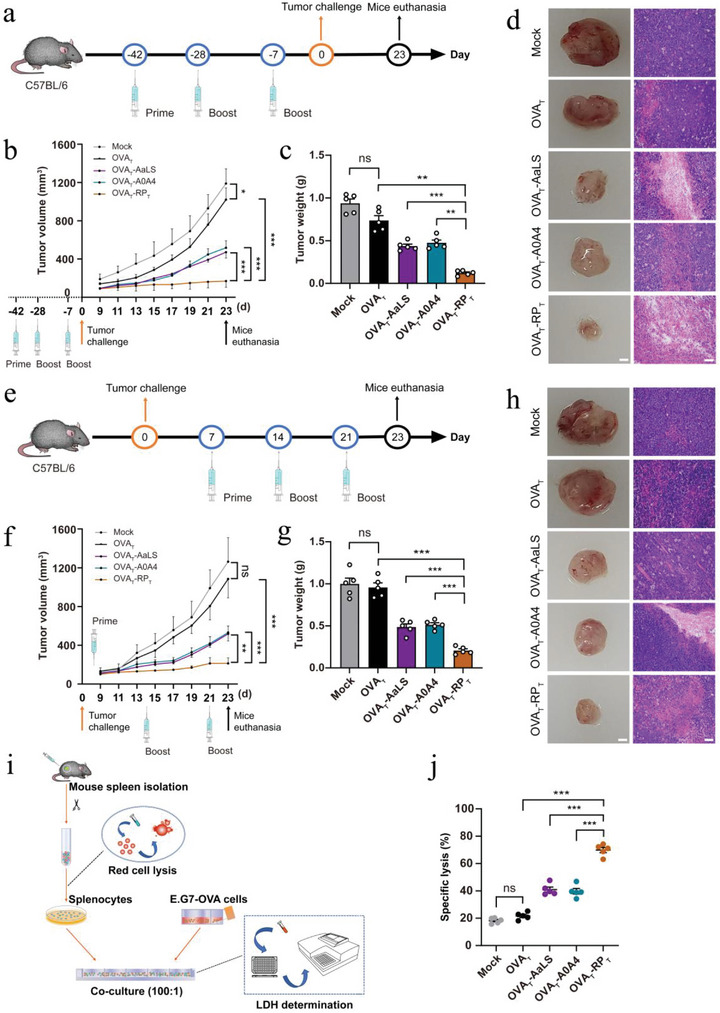
Antitumor immunity and direct tumor killing activities of RP_T_‐derived tumor nanovaccines. a) Schematic diagram of the prophylactic immune study of tumor‐bearing mice. b) Average tumor growth curve of mice was measured every other day until day 23, and tumor volume was calculated using the formula 0.5 × length × width^2^ (*n* = 5). c) Tumor wet weight of different prophylactic groups on day 23 (*n* = 5). d) Tumor photos and H&E‐stained tumor sections of different prophylactic groups. Scale bar: 2 mm (tumor photos) or 100 µm (H&E‐stained sections). e) Schematic diagram of the therapeutic immune study of tumor‐bearing mice. f) Average tumor growth curve of mice was measured every other day until day 23, and tumor volume was calculated using the formula 0.5 × length × width^2^ (*n* = 5). g) Tumor wet weight of different therapeutic groups on day 23 (*n* = 5). h) Tumor photos and H&E‐stained tumor sections of different therapeutic groups. Scale bar: 2 mm (tumor photos) or 100 µm (H&E‐stained sections). i) Schematic diagram and j) quantification of the specific killing of E.G7‐OVA tumor cells in vitro (*n* = 5). Data are expressed as the mean ± SEM. *p < 0.05, **p < 0.01, ***p < 0.001; ns represents not significant.

We constructed in vivo and in vitro tumor models to examine the inhibitory effect of RP_T_‐derived nanovaccine immunization on tumor growth. First, we established an in vivo prophylactic tumor model (**Figure** **5**a). In this model, C57BL/6 mice were first immunized with three doses of OVA_T_‐RP_T_ or other controls and then subcutaneously inoculated with E.G7‐OVA tumor cells, followed by the measurement of tumor growth. Free OVA_T_ hardly offered any significant tumor inhibition compared to the mock group, while the three nanovaccines conferred considerable immune protection, among which OVA_T_‐RP_T_ achieved the maximum prophylactic efficacy, with a tumor inhibition rate of 85.72% on day 23 post‐tumor challenge (Figure 5b). In the therapeutic tumor model, C57BL/6 mice were subcutaneously inoculated with E.G7‐OVA. Seven days later, the mice were immunized with three doses of OVA_T_‐RP_T_ or other controls (Figure 5e), followed by monitoring the changes in tumor size. Like the prophylactic model, OVA_T_‐RP_T_ exhibited the most effective tumor inhibition, with a tumor suppression rate of 83.09% on day 23 post‐tumor challenge (Figure 5f). The tumor weight in each group showed the same tendency (Figure 5c,g). The photos and hematoxylin and eosin (H&E) staining of resected tumors further proved the superior tumor suppression of OVA_T_‐RP_T_ (Figure 5d,h). The weights of the mice did not change significantly (Figure S7a,b, Supporting Information). H&E staining of other major organs showed negligible side effects for all treatments (Figure S7c, Supporting Information). In addition, creatinine (CRE), uric acid (UA), blood urea nitrogen (BUN), glutamic pyruvic transaminase (GPT), and glutamic oxaloacetic transaminase (GOT) were measured to assess the systemic cytotoxicity of the different treatment groups (Figure S7d, Supporting Information). No abnormal indicators were observed in any of the groups.

Next, the specific killing of tumor cells by T cells stimulated with OVA_T_‐RP_T_ was verified in an in vitro co‐culture model (Figure 5i). Splenocytes isolated from immunized mice were co‐incubated with E.G7‐OVA tumor cells at a ratio of 100:1. The lactate dehydrogenase levels in the culture supernatant were measured to calculate apoptosis or necrosis of tumor cells. As shown in Figure 5j, splenocytes from OVA_T_‐RP_T_ immunized mice killed approximately 69.90% of tumor cells, which was higher than the killing rate of 40.96% for OVA_T_‐AaLS and 39.72% for OVA_T_‐A0A4. Unfortunately, free OVA_T_ did not offer significant tumor killing compared to the mock group. Altogether, these results clearly indicated that SC‐RP_T_‐derived nanovaccines could induce robust antitumor immunity and direct tumor killing.

### 2.6 RP_T_‐Derived SARS‐CoV‐2 Nanovaccines Drive Antigen‐Specific CD8^+^ T Cell‐ and CD4^+^ T Helper 1 (Th1)‐Biased Responses

**Figure 6:**
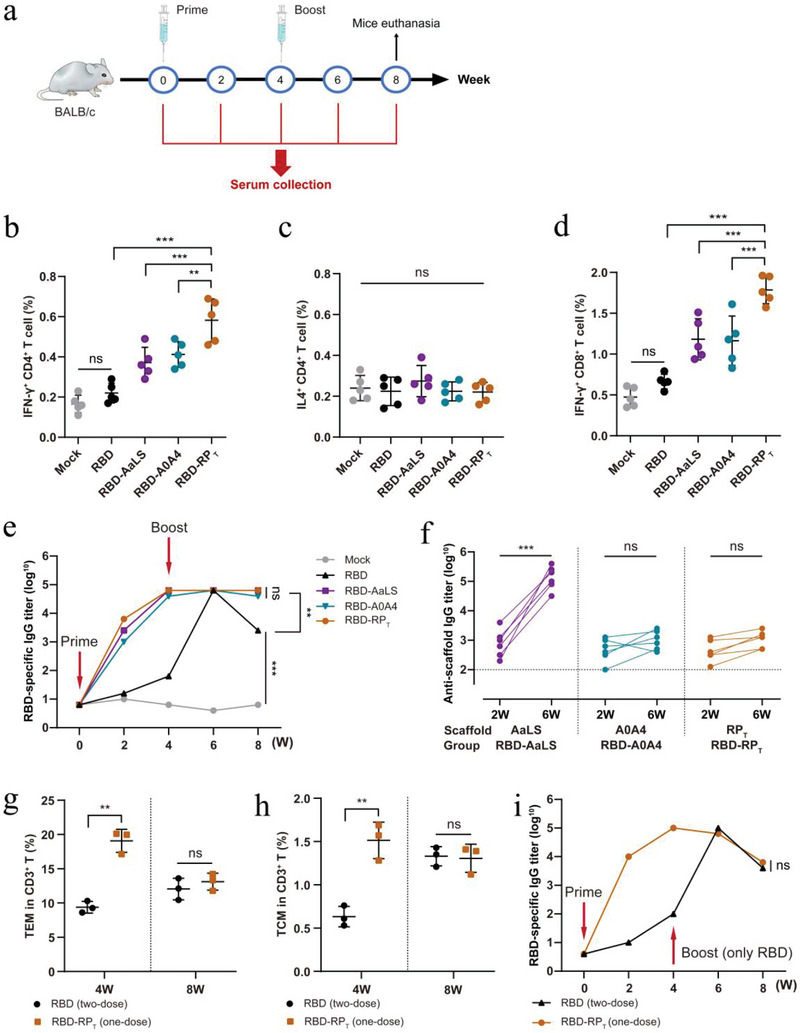
Antigen‐specific CD8^+^ T cell‐ and Th1‐biased responses of RP_T_‐derived SARS‐CoV‐2 nanovaccines. a) Schematic of BALB/c mice vaccination. Mice were prime/boost‐vaccinated with different vaccines at weeks 0 and 4. Serum was collected every two weeks. Mice were euthanized at week 8. b) Quantification of the percentage of IFN‐*γ* ^+^ CD4^+^, c) IL‐4^+^ CD4^+^, and d) IFN‐*γ* ^+^ CD8^+^ T cells in the spleen of mice at 8 weeks post‐immunization (*n* = 5). e) RBD‐specific IgG titers were calculated and plotted as a time‐course curve (*n* = 6). f) Scaffold‐specific IgG titers of mice at 2 and 6 weeks post‐immunization (*n* = 6). g) Quantification of the percentage of TEM and h) TCM in the spleen of mice at 4 and 8 weeks post‐immunization (*n* = 3). i) RBD‐specific IgG titers were calculated and plotted as a time‐course curve in a single‐dose immune model (*n* = 6). BALB/c mice in the RBD‐RP_T_ group were subcutaneously immunized with RBD‐RP_T_ only at week 0, and mice in the free RBD group were immunized with RBD at weeks 0 and 4. Data are expressed as the mean ± SEM. *p < 0.05, **p < 0.01, ***p < 0.001; ns represents not significant.

Several studies have shown that the T cell response is a critical component of immune protection against SARS‐CoV‐2, which may constitute the second line of defense in addition to neutralizing antibodies.^[^ ^30^, ^31^, ^32^ ^]^ Developing a T cell immunity‐based vaccine can significantly protect against SARS‐CoV‐2 infection in vaccinated individuals. We further constructed an RBD‐based SARS‐CoV‐2 nanovaccine (RBD‐RP_T_) with T cell‐activating SC‐RP_T_, as shown in Figure 2a. BALB/c mice were subcutaneously immunized with free RBD, RBD‐AaLS, RBD‐A0A4, and RBD‐RP_T_ in a prime‐boost manner (**Figure** **6**a). All vaccines were formulated with the AddaVax adjuvant. Equal volumes of adjuvant‐vaccinated mice were set as the mock group. To verify that RBD‐RP_T_ also induces potent T cell responses, we analyzed different T cell populations in the spleens of the vaccinated mice. At four weeks post‐boost vaccination, RBD‐RP_T_ elicited more CD4^+^ and CD8^+^ effector T cells (CD62L^low^ CD44^high^ TEM) and central memory T cells (CD62L^high^ CD44^high^ TCM) than the other controls (Figure S8a–d,g, Supporting Information). Previous studies on SARS‐CoV‐2 have shown that Th2‐skewed CD4^+^ T cells are related to the induction of vaccine‐associated enhanced respiratory disease, while Th1‐biased immune responses enhance protection against viral infection.^[^ ^33^, ^34^ ^]^ RBD‐RP_T_ provoked more Th1‐biased IFN‐*γ* ^+^ CD4^+^ T cells than other controls, whereas the percentages of Th2‐skewed IL‐4^+^ CD4^+^ T cells did not differ across the different groups (Figure 6b,c). Additionally, RBD‐RP_T_ induced more antigen‐specific and polyfunctional CD8^+^ T cells to secrete IFN‐*γ*, IL‐2, and TNF‐*α* to elicit immediate antiviral effects during pathogen encounters (Figure 6d; Figure S8e,f, Supporting Information). We periodically detected RBD‐specific antibody levels in the peripheral blood of mice. The titers of RBD‐specific antibodies in the nanovaccine groups, especially in the RBD‐RP_T_ group, peaked at four weeks post‐priming, indicating that SC‐RP_T_ notably enhanced the immunoreactivity of the RBD antigen (Figure 6e). In line with several previous studies, we detected high titers of antibodies against SC‐AaLS in the RBD‐AaLS group, whereas the antibody levels of SC‐RP_T_ in the RBD‐RP_T_ group and SC‐A0A4 in RBD‐A0A4 group were negligible (Figure 6f; Figure S8h, Supporting Information).

Developing a single‐dose vaccine for the rapid onset of immune responses could protect individuals at acute risk during virus transmission. We further evaluated the immune responses induced by a single‐dose of RBD‐RP_T_. Compared with the free RBD group immunized in a prime‐boost manner, RBD‐RP_T_ triggered a higher proportion of TEM (CD3^+^ CD44^high^ CD62L^low^) and TCM (CD3^+^ CD44^high^ CD62L^high^) at four weeks post‐one‐dose of the vaccine and maintained high levels at week 8 (Figure 6g,h). Meanwhile, according to the detection of RBD‐specific antibody titer, the antibody level of the single‐dose RBD‐RP_T_ group was slightly attenuated after the 4th week and was equal to that of the two‐dose free RBD group in the 8th week, with no statistically significant difference (Figure 6i). These results indicated that the RP_T_‐derived nanovaccine could induce potent and safe T cell immune responses and maintain humoral immune activation with low anti‐scaffold antibody response, making it a candidate for developing a rapid single‐dose antiviral vaccine.

### 2.7 RP_T_ Drives Type‐1 Conventional DCs (cDC1s) Differentiation to Mediate Specific T Cell Immune Responses

**Figure 7:**
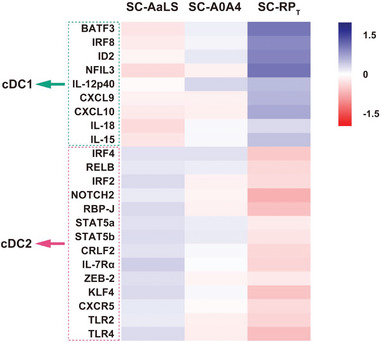
Gene heat map of SC‐AaLS‐, SC‐A0A4‐ and SC‐RP_T_‐pulsed BMDCs.

In vaccinology, gene expression induced by initiating APCs may be utilized to predict antigen immunogenicity and T cell activation types, thereby aiding in understanding the immune mechanisms of vaccination. We identified gene expression signatures induced by SC‐RP_T_ in APCs to elucidate the molecular mechanisms that account for SC‐RP_T_‐primed immune responses. We performed differentiation gene expression profile analysis on mouse immature bone marrow dendritic cells (BMDCs) cultured with pure SC‐AaLS, SC‐A0A4, and SC‐RP_T_ to explore the impact of scaffold‐mediated DC differentiation on T cell activation (**Figure** **7**). cDC1s in concert with *γδ* T cells, natural killer T cells, natural killer cells, and other innate lymphoid cells (ILCs) activate CD8^+^ cytotoxic T lymphocytes and Th1 cells, whereas transcription factor Krüppel‐like factor 4 (KLF4)‐dependent type‐2 conventional DCs (cDC2s) in concert with ILCs, *γδ* T cells, basophils, or other innate cells drive Th2 cell responses.^[^ ^35^ ^]^ SC‐RP_T_ upregulated the expression of genes related to cDC1s, including transcription factors BATF3, interferon regulatory factor 8 (IRF8), inhibitor of DNA‐binding 2 (ID2), NFIL3, chemokine CXC‐chemokine ligand 9/10 (CXCL9/10), and cytokines IL‐12p40/18/15, and downregulated the expression of genes related to cDC2s, including transcription factors IRF4/2, RELB, ZEB2, KLF4, NOTCH2/RBP‐J signal axis, signal transducer and activator of transcription 5a/b (STAT5a/b)/CRLF2/IL‐7R*α* signal axis, chemokine receptor CXCR5 (C‐X‐C motif chemokine receptor 5), and toll‐like receptors TLR2/4. However, SC‐AaLS seemed to slightly upregulate the expression of genes related to cDC2s and downregulate the expression of genes related to cDC1s. No evidence was observed in DCs differentiation induced by SC‐A0A4. These results suggest that SC‐RP_T_ may induce DCs to differentiate into cDC1s to prime CD8^+^ cytotoxic T lymphocytes and Th1 responses, assisting in antitumor/viral infection of antigens.

## 3 Discussion

For clinical applications, the usefulness of antigen delivery vehicles depends on a) conferring high stability to antigens during formulation, storage, transport, and administration; b) conferring high immunogenicity to antigens and no immunotoxicity; c) low anti‐vehicle antibody generation to avoid the premature clearance of antigen‐vehicle complex; d) simple and rapid production. In this study, an ab initio designed nanoscaffold RP_T_ derived from AaLS showed various positive features for effective antigen delivery compared to AaLS, such as a) robustness to heating, freeze‐thawing, lyophilization, strong acid–base, and high salt concentration; b) efficient elicitation of T cell‐mediated immunity with high safety; c) low anti‐scaffold antibody generation; d) simple production in *E. coli* with excellent protein yield, potentially facilitating T‐cell immunity‐dependent vaccines development.

Organic or inorganic nanomaterials usually have two functions in vaccine applications: an efficient antigen delivery carrier or an adjuvant to stimulate the immune system. Inorganic nanomaterials are toxic and non‐biodegradable.^[^ ^4^ ^]^ Protein‐based nanoparticles are now recognized as suitable materials for new developments in nanotechnology.^[^ ^36^ ^]^ The benefits of atomically precise assembly endow these particle surfaces with natural pathogen‐associated molecular patterns resembling virus particles, which are readily identified by the immune system and trigger immune activation. Nevertheless, this unregulated activation of the innate immune system may result in immunotoxicity and/or premature clearance of the antigen‐scaffold complex. Controlling the interaction between nanoparticles and the immune system is crucial for the safe advancement of nanotechnology, especially for biomedical applications. Previous studies have tailored the immune system recognition of nanoparticles by altering their size,^[^ ^37^ ^]^ shape,^[^ ^38^ ^]^ or surface charge.^[^ ^39^ ^]^ Fettelschoss‐Gabriel et al. have inserted the peptide TT_830‐843_ from the tetanus toxin, a powerful universal T cell‐activating epitope, into VLPs to elicit T cell activation and facilitate vaccination.^[^ ^40^ ^]^ Few studies have shown that specific immune types can be activated by changing the antigenic properties of nanoparticles. In this study, RP_T_ in rational design markedly upregulated the gene expression of transcription factors and cytokines related to the differentiation of cDC1s, which promoted the cross‐presentation of antigens to CD8^+^ T cells and Th1 polarization of CD4^+^ T cells with low anti‐scaffold antibody generation.

Different studies have reached inconsistent conclusions regarding whether anti‐scaffold antibody responses detract from or compete with antigen‐specific reactions. A recent study by Ma et al. found that mice subjected to prime‐boost immunization with a nanoparticle vaccine prepared by covalently conjugating the self‐assembled 24‐mer ferritin to the RBD and/or heptad repeat subunit of the SARS‐CoV‐2 spike protein, and the high titers of ferritin‐specific antibodies did not impair RBD‐ and HR‐specific antibody levels.^[^ ^11^ ^]^ Marcandalli et al. found that pre‐existing immunity against the I53‐50 scaffold did not deleteriously affect the antigen‐specific response, even after multiple boosts.^[^ ^41^ ^]^ However, they noted that further research, preferably in people, is necessary to completely comprehend the role of antiscaffold reactions. Another study found that a multi‐antigen vaccine developed by fusion of a linear B cell epitope (PB10) from the ricin toxin with the C‐terminus of LS, an icosahedral symmetry capsid derived from *Bacillus anthracis*, had an intense immune response against the scaffold than a conjugated epitope (PB10) in all immunized mice, resulting in an immunodominant effect of LS that may somehow suppress immunogenicity of the epitope, which may partly explain the suboptimal neutralizing antibody titers to the epitope after three vaccinations.^[^ ^42^ ^]^ Okba et al. immunized rabbits with RBD‐AaLS, multimeric RBD‐presenting particles produced by covalently coupling RBD to AaLS particles, to elicit antibody responses against AaLS in all groups.^[^ ^13^ ^]^ They also developed a heterologous scaffold strategy using AaLS and I3 to reduce anti‐scaffold responses. An AaLS‐based vaccine for human immunodeficiency virus completed a phase I clinical trial (NCT03547245), demonstrating the availability of this antigen delivery platform. Nonetheless, we eliminated the introduction of B cell epitopes in the RP_T_ computational design to minimize anti‐scaffold antibody responses.

Using two disease models, this study thoroughly verified the robustness and T cell activation of RP_T_‐based nanovaccines. Given the ease of treatment in OVA‐expressing tumors, we analyzed whether pure RP_T_ simultaneously promoted antigen presentation and stimulated the antitumor immune response in the absence of adjuvants. The immune effect of the SARS‐CoV‐2 vaccine showed that RP_T_ could synergize with adjuvants to elicit T cell‐mediated immunity. Evaluating the longevity of vaccine‐induced immune responses is warranted. In post‐prime vaccination, the RP_T_‐derived nanovaccine elicited a potent T cell‐based immune response and maintained it for a long time. Owing to the robust immune response induced after one dose, the RP_T_‐based nanovaccine could be a candidate for developing a rapid single‐dose antitumor/viral vaccine based on T cell immunity. Nevertheless, our research has certain limitations: i) no live virus challenge was performed against the SARS‐CoV‐2 vaccine assessment, which will be our next step; and ii) at this stage, SC‐AaLS, SC‐A0A4, and SC‐RP_T_ all demonstrated proper safety, whereas the nanoscaffold with tailored antigen properties is critical for immune activation controllability and long‐term vaccine safety. Our future investigation will also include monitoring the biochemical index changes following the inoculation of these nanoscaffolds.

## Conflict of Interest

The authors declare no conflict of interest.

## Supporting information

Supporting Information

## Data Availability

The data that support the findings of this study are available from the corresponding author upon reasonable request.
