# Supplementary material for: T Cell Activating Thermostable Self‐Assembly Nanoscaffold Tailored for Cellular Immunity Antigen Delivery
Source: Adv Sci (Weinh). 2023 Jul 3;10(26):2303049. doi: 10.1002/advs.202303049 (PMC10502629; doi:10.1002/advs.202303049)
Supplement: Supplementary file 1 — Supporting Information [file ADVS-10-2303049-s001.pdf]

ADVANCED SCIENCE
Open Access

Supporting Information

for *Adv. Sci.*, DOI 10.1002/advs.202303049

T Cell Activating Thermostable Self-Assembly Nanoscaffold Tailored for Cellular Immunity Antigen Delivery

Jinsong Zhang, Jianghua Yang, Qianlin Li, Ruihao Peng, Shoudong Fan, Huaimin Yi, Yuying Lu, Yuanli Peng, Haozhen Yan, Lidan Sun, Jiahai Lu and Zeliang Chen**

Supplementary materials and methods

Reagents

FITC-conjugated anti-mouse CD3, PerCP/Cyanine5.5-conjugated anti-mouse CD4, PE/Cyanine7-conjugated anti-mouse CD8a, PE-conjugated anti-mouse CD69, APC-conjugated anti-mouse CD45R/B220, APC-conjugated anti-mouse CD11c, PE-conjugated anti-mouse MHCII, APC-conjugated anti-mouse CD44, PE-conjugated anti-mouse CD62L, APC-conjugated anti-mouse IFN-γ, PE-conjugated anti-mouse IL-4, APC-conjugated anti-mouse H-2K^b bound to SIINFEKL, APC-conjugated anti-mouse CD40 antibodies, and mouse cytokine (IFN-α, IL-12p70, and IFN-γ) ELISA kits were purchased from BioLegend (San Diego, USA). Anti-mouse CD3, anti-mouse CD11c, anti-mouse CD80, anti-mouse CD86 antibodies, PE/Cyanine5.5, and the APC conjugation kit lightning link were purchased from Abcam (Cambridge, UK). Goat anti-mouse secondary antibodies and granulocyte-macrophage colony-stimulating factor (GM-CSF) were purchased from Thermo Fisher Scientific (Waltham, USA). The lactate dehydrogenase (LDH) cytotoxicity assay kit was purchased from Beyotime (Shanghai, CN). Uric acid (UA), urea nitrogen (BUN), glutamate pyruvic transferase (GPT), and glutamic oxaloacetic transaminase (GOT) assay kits were purchased from Beijing Boxbio Science & Technology Co., Ltd. (Beijing, CN). The serum creatinine (CRE) assay kit was purchased from Suzhou Grace Biotech Co. Ltd. (Suzhou, CN). Isopropyl β-D-1-thiogalactopyranoside (IPTG) and the Co-NTA agarose resin were purchased from Takara (Tokyo, JPN). Fetal bovine serum (FBS) was purchased from Corning (NY, USA). Dulbecco’s modified

Eagle's medium (DMEM) and Roswell Park Memorial Institute (RPMI) 1640 medium were purchased from Gibco (NY, USA). Other chemical reagents were purchased from Beijing Tiangen Biotech Co. Ltd. (Beijing, CN).

Cells and animals

The murine dendritic cell line, DC2.4, purchased from ATCC (Manassas, USA), was cultured in DMEM containing 10% FBS. E.G7-OVA tumor cells, purchased from ATCC, were cultured in RPMI 1640 containing 10% FBS. B3Z T cells were cultured in RPMI 1640 containing 10% FBS from the Nilabh Shastri Laboratory, University of California, Berkeley, USA. Cells were incubated at 37 °C in a humidified atmosphere containing 5% CO_2.

C57BL/6 and BALB/c mice were purchased from Beijing Hfk Bioscience Co. Ltd. (Beijing, CN). All mice were raised under specific pathogen-free conditions in an animal facility and received care in compliance with the guidelines outlined in the Guide for the Care and Use of Laboratory Animals. All procedures were approved by the Laboratory Animal Ethics Committee of the School of Public Health at Sun Yat-sen University (Approval Number: 2022-029).

Immunoinformatics prediction and computational modeling of T cell-activating nanoscaffolds

The primary amino acid sequence of the template AaLS subunit was retrieved from the *UniProt Knowledgebase* (accession number: O66529). *Protein BLAST* was performed to search native homologous sequences from thermophiles with a spatial structure similar to AaLS. Due to the lack of experimental 3D structures of

homologous sequences in the *Protein Data Bank* (https://www.rcsb.org/), three-dimensional structures of these sequences were generated via homology modeling of the *Swiss Model* (https://swissmodel.expasy.org/) based on the known X-ray crystal structure of AaLS. Ten native sequences with the same spatial structure as AaLS were screened. The antigenicity of homologous sequences was further analyzed using *VaxiJen v 2.0 Server* (http://www.ddg-pharmfac.net/vaxijen/VaxiJen/VaxiJen.html). Bacteria were selected in "*TARGET ORGANISM*", and the threshold was set to 0.4 to exclude sequences with poor antigenicity.

MHCI molecule-binding epitopes of homologous sequences were predicted using *NetMHCpan EL 4.1* (http://tools.iedb.org/mhci/) of *IEDB* and *NetMHC-4.0 Server* (https://services.healthtech.dtu.dk/service.php?NetMHC-4.0). The HLA subtypes were selected to cover approximately 97% of the MHCI molecule-binding epitopes of the world population. The peptide length was set at 9. *VaxiJen v2.0 Server* performed an antigenicity analysis of the predicted overlapping epitopes. Bacteria were selected in "*TARGET ORGANISM*", and the threshold was set to 0.4 to obtain MHCI-dominant epitopes. MHCII molecule-binding epitopes of homologous sequences were predicted using *NetMHC II-2.3 Server* (https://services.healthtech.dtu.dk/service.php?NetMHCII-2.3) and *NetMHC II pan-4.0 Server* (https://services.healthtech.dtu.dk/service.php?NetMHCIIpan-4.0). The HLA subtypes were selected to cover approximately 99% of the MHCII molecule-binding epitopes of the world population. The peptide length was set at 15.

VaxiJen V 2.0 Server performed antigenicity analysis of overlapping epitopes to identify MHCII-dominant epitopes.

The T-dominant epitopes substituted the sequences at the same position of the AaLS subunit, and some amino acids were mutated appropriately to maintain the structural stability of the reassembled sequence. *VaxiJen V 2.0 Server* performed antigenicity analysis, and *ProtParam Tool* (https://web.expasy.org/protparam/) performed stability analysis of the reassembled sequences. An antigenicity score greater than 0.4000 classifies the protein as “*Probable ANTIGEN*”. An instability index less than 40.00 classifies the protein as stable (https://web.expasy.org/protparam/). We excluded reassembled sequences with poor antigenicity and spatial structure quality. The 3D structures of the reassembled sequences were generated by homology modeling using the *Swiss Model.* The sequences with the same 3D structure as AaLS were identified as reassembled proteins (RPs).

Protein expression and purification

SpyCatcher (SC)-nanoparticle expression constructs, consisting of SC (VTTLSGLSGEQGPSGDMTTEEDSATHIKFSKRDEDGRELAGATMELRDSSGK TISTWISDGHVKDFYLYPGKYTFVETAAPDGYEVATPIEFTVNEDGQVTVDGE ATEGDAHT), $(GGS)_4$ spacer, primary amino acid sequence of nanoparticle proteins, GS spacer, and C-terminal 6× His tag, were synthesized by Shanghai Sangon Biotech Co., Ltd. (Shanghai, CN) and subcloned into a *pET28a* expression plasmid. FITC-tagged OVA_T genetically fused to SpyTag (ST) at the N-terminus was

synthesized and expressed by Shanghai Sangon Biotech Co., Ltd. RBD (SARS-CoV-2 spike protein residues 319–541) genetically fused to ST at the N-terminus was synthesized by Shanghai Sangon Biotech Co., Ltd. and subcloned into a *pcDNA3.1* expression plasmid. The *pcDNA3.1*-ST-RBD was transformed into *HEK293T* cells and expressed by Shenzhen AIVD Biotech Co., Ltd. (Shenzhen, CN).

The *pET28a*-SC-nanoparticle was transformed into *E. coli* BL21 cells and were grown on LB-Agar plates (50 μg/mL kanamycin) for 16 h at 37 °C. A single clone was amplified in the LB medium (50 μg/mL kanamycin) and incubated at 37 °C with shaking at 200 rpm. At an A_{600} of 0.8, cultures were induced with IPTG (0.5 mM) and grown for 18 h with shaking at 200 rpm at 22 °C. The culture-derived pellets were harvested and lysed by sonication before centrifugation at 15000 rpm for 15 min at 4 °C. The supernatant was incubated with Co-NTA agarose resin to enrich His-tagged SC-nanoparticles, and the target protein was eluted with Imidazole-containing Tris buffer. The purified protein was concentrated, followed by buffer replacement with conventional Tris buffer. Endotoxins were removed from the samples using Triton X-114. The concentration of endotoxin-depleted particles was determined using the BCA assay and stored at -20 °C.

Nanovaccine conjugation and purification

SC-nanoparticles (number of subunits) were reacted with 1.5× molar excess of ST-OVA_T or ST-RBD at 4 °C for 16 h in a neutral TBS buffer. Conjugated OVA_T- or RBD-nanoscffolds were separated from free OVA_T or RBD via size exclusion chromatography (SEC) and concentrated with an ultrafiltration device to collect

nanovaccines. The concentration of nanovaccines was measured using a BCA assay. SEC, SDS-PAGE with Coomassie staining, and negatively stained transmission electron microscopy (TEM) were used to characterize the particle purity and homogeneity.

Transmission electron microscopy (TEM)

TEM of nanoparticles was carried out using negative-stain electron microscopy at Shanghai WEIPU Testing Technology Group Co., Ltd. (Shanghai, CN). Briefly, nanoparticles (0.2 mg/mL) were applied to glow-discharged carbon 200 mesh copper grids covered with a thin layer of continuous film. Samples were stained with 2% uranyl acetate, followed by imaging using a Hitachi TEM System HC-1 (Tokyo, JPN) operating at an acceleration voltage of 120 kV. Images were recorded at a magnification of 200,000×.

Differential scanning calorimetry (DSC)

The melting temperatures were measured using a Netzsch DSC 200 F3 (SELB, GER). The equipment (without any sample or reference) was run for 30 min to ensure the accuracy of the experimental results. The test sample (1 mg/mL) was placed in a crucible sample tank and measured from room temperature to 160 °C at a scan rate of 2.0 K/min to obtain the melting curves. Each sample was scanned twice. DSC measurement data were analyzed using Microsoft Excel software.

Dynamic light scattering (DLS)

The samples were centrifuged for 30 min at 15000 rpm at 4 °C to remove any aggregates. Before each measurement, a quartz cuvette was incubated in the

instrument for 5 min to stabilize the sample temperature. The protein concentration of the sample was controlled at approximately 0.2 mg/mL. Briefly, 30 µL of the sample was measured at 25 °C using a Malvern Zetasizer Nano ZS (Malvern, UK). The intensity of the size distribution was normalized to the peak value and plotted using GraphPad Prism 8.0.

Stability tests

After heating, multiple cycles of freeze-thawing, and lyophilization, nanoparticle proteins were centrifuged at 15,000 rpm for 30 min at 4 °C to remove any aggregates, and stability analysis was performed as previously described.[1] For the acid and alkaline treatments, samples were incubated in PBS at pH (0.1, 4, 7, and 14) at 37 °C for 24 h. The solutions were neutralized prior to sample analysis. For treatment with salt ions, samples were incubated in PBS containing salt (0, 50, 100, and 500 mM) at 37 °C for 48 h. The salt solutions were replaced with conventional solutions before sample analysis. The solubility and integrity of the nanoparticles were detected using SDS-PAGE with Coomassie staining and DLS.

Flow cytometry

Mouse inguinal lymph nodes or spleens were collected in PBS containing 2% FBS, ground on a 200-mesh filter, and incubated in ACK lysis buffer to remove red blood cells. The remaining cells were centrifuged at 1500 rpm for 10 min, and the cell pellet was resuspended in 20–100 µL PBS to prepare a single-cell suspension. The cell concentration was adjusted to 1×10^7 cells/mL in PBS containing 0.5% FBS for flow cytometric analysis. Cells were blocked with 3% FBS in PBS for 15 min and

further stained with indicated fluorochrome-conjugated monoclonal antibodies for 30 min at 4 °C. Cells were centrifuged to remove unbound antibodies and resuspended for detection using a Beckman CytoFLEX S (Brea, USA).

***In vitro* cellular uptake, APC activation and cross-presentation**

DC2.4 cells were seeded in 24-well plates at 2.5×10^5 cells/well and incubated for 12 h. The night before treatment, the culture medium was replaced with a medium containing 2% FBS. Cells were then treated with a complete medium containing FITC-tagged OVA_T, OVA_T-AaLS, OVA_T-A0A4, and OVA_T-RP_T (OVA_T concentration: 1 μg/mL) for 12 h at 37 °C in a 5% CO_2 atmosphere. Complete medium served as the negative control. Cells were fixed with 4% paraformaldehyde for 30 min before being washed three times with cold PBS. Cell fluorescence intensity was detected using flow cytometry and immunofluorescence staining to assess cellular uptake capacity.

DC2.4 cells were seeded in 6-well plates at 1.2×10^6 cells/well and then incubated with OVA_T, OVA_T-AaLS, OVA_T-A0A4, and OVA_T-RP_T (OVA_T concentration: 1 μg/mL) at 37 °C for 24 h. Complete medium served as the negative control. Cell culture supernatants were collected and centrifuged at 4500 rpm for 5 min at 4 °C. The amounts of IFN-α and IL-12p70 in the supernatant were measured using ELISA kits, according to the manufacturer's instructions. The cells were stained with APC-conjugated CD40, CD80, and CD86 monoclonal antibodies for 30 min at 4 °C before flow cytometric analysis. The expression levels of these cytokines were measured to assess APC activation.

The *in vitro* cross-presentation of OVA_T was assessed using the B3Z antigen

presentation system. B3Z T cells, $CD8^+$ T cell hybridomas, express a TCR that specifically recognizes OVA_T (SIINFEKL) in the context of $H\text{-}2K^b$ and produces β-galactosidase. DC2.4 cells were seeded in 96-well plates at 1×10^5 cells/well for 12 h. OVA_T, OVA_T-AaLS, OVA_T-A0A4, and OVA_T-RP_T (OVA_T concentration: 1 µg/mL) were added and incubated for 24 h at 37 °C. Complete medium served as the negative control. The culture supernatant was discarded, and the cells were washed twice with PBS. The concentration of B3Z T cells was adjusted to 1×10^6 cells/mL, and 100 µL of cells was added and co-cultured with DC2.4 cells for 24 h. Cells were collected by centrifugation at 1500 rpm and washed twice with PBS. The cells were resuspended in 150 µL of CPRG buffer (1×PBS supplemented with 0.15 mM chlorophenol red-β-$_D$-galactopyranoside (CPRG), 100 µM β-mercaptoethanol, 0.1 % Triton X-100, and 9 mM $MgCl_2$) until the color reaction had progressed sufficiently. One hundred microliters of sample were transferred to 96-well plates, and the absorbance of released chlorophenol red was measured at a wavelength of 590 nm using a plate reader.

Prophylactic and therapeutic tumor models

For the prophylactic tumor model, C57BL/6 mice were immunized with a 10-µg dose of OVA_T nanovaccines on days -42, -28, and -7, followed by the subcutaneous inoculation of 1.0×10^6 E.G7-OVA log-phase tumor cells in the right axilla on day 0. Equal volumes of saline-injected mice were used as the mock group. Tumor volumes were measured using Vernier calipers, and the mice were weighed every other day. Tumor volume was calculated according to the formula: $V = 0.5\times\text{length}\times\text{width}^2$.

Mice were euthanized on day 23 post-tumor inoculation, and tumor tissues were collected for wet weighing and pathological examination. Briefly, tumor sections were prepared for hematoxylin and eosin (H&E) staining to observe the histopathological morphology of each group.

C57BL/6 mice were subcutaneously inoculated with 1.0×10^6 E.G7-OVA log-phase tumor cells in the right axilla and then immunized with a 10-μg dose of OVA_T nanovaccines on days 7, 14, and 21 to establish a therapeutic tumor model. Equal volumes of saline-injected mice were used as the mock group. The tumor volume and body weight of the mice were measured regularly. Mice were euthanized on day 23 post-tumor inoculation, and tumor tissues were collected for wet weighing and pathological examination.

***In vitro* tumor cell-specific killing model**

Disruption of the cell membrane structure caused by apoptosis or necrosis results in the release of enzymes from the cytoplasm into the culture medium. Lactate dehydrogenase activity is stable, and its release is regarded as an important indicator of cell membrane integrity and is widely used for cytotoxicity detection. Briefly, three doses of immunized mice were euthanized to collect spleens, and a single-cell suspension was prepared after the removal of red blood cells. The collected splenocytes (1.0×10^5 cells/well) and E.G7-OVA tumor cells (1.0×10^3 cells/well) were seeded in 48-well plates and cultured at 37 °C in a 5% CO_2 atmosphere. After 24 h, supernatants were collected by centrifugation, and apoptosis of tumor cells was detected using a lactate dehydrogenase cytotoxicity assay kit according to the

manufacturer's instructions.

***In vivo* cytotoxicity assay**

Serum levels of liver function indexes, GPT and GOT, and renal function indexes, CRE, UA, and BUN were measured to assess systemic cytotoxicity. All the testing procedures were performed using commercial detection kits in accordance with the manufacturer's instructions.

SARS-CoV-2 nanovaccine immune model

For the prime-boost immune model, BALB/c mice were subcutaneously immunized with a 10-μg dose of RBD nanovaccines formulated with AddaVax™ adjuvant (Invivogen, San Diego, USA) at weeks 0 and 4. Equal volumes of adjuvant-vaccinated mice were used as the mock group. For the single-dose immune model, BALB/c mice in the RBD-RP_T group were subcutaneously immunized with RBD-RP_T only at week 0, and mice in the free RBD group were immunized with RBD at weeks 0 and 4. Serum was collected every two weeks. Mice were euthanized at week 8, and their spleens were collected for related index analysis.

Antibody titer detection

Immunized animal serum was collected via centrifugation to detect specific antibody titers against different antigens. Antigen proteins were coated onto high-binding 96-well plates at a concentration of 1 μg/mL and incubated overnight at 4 °C. The 96-well plates were washed three times with PBS/T (PBS containing 0.05% Tween 20) and blocked overnight with 5% BSA solution at 4 °C. Serially diluted serum (10^2, 10^3, 10^4, 10^5, 10^6, 10^7, and 10^8 dilutions) was added to each well in

duplicate and incubated at 37 °C for 0.5–1 h. After washing, horseradish peroxidase (HRP)-conjugated goat anti-mouse antibody was added at a dilution of 1:20000 and incubated for another 0.5 h. After washing the plates again, TMB solution was added, and the reaction was stopped with a stop solution after sufficient color development. The absorbance was measured at a wavelength of 450 nm using a plate reader. The end-point titer was defined as the dilution whose absorbance value was equal to twice the mean of the background (negative serum).

Gene heat map analysis

Bone marrow-derived dendritic cells (BMDCs) were prepared as previously described.[2] Briefly, bone marrow cells were collected by flushing tibias of C57BL/6 mice with complete medium (RPMI 1640 supplemented with 50 μM 2-mercaptoethanol, 2 mM L-glutamine, 100 U/mL penicillin, 100 μg/mL streptomycin, and 10% FBS). Cells were seeded in 6-well plates and treated with a complete medium containing 200 U/mL of GM-CSF for 7 days at 37 °C in a 5% CO_2 atmosphere. Immature BMDCs were collected and incubated for 24 h with 50 μg/mL of SC-AaLS, SC-A0A4, and SC-RPT at a final concentration of 2.5×10^6 cells/mL.

Total RNA was extracted from cells using TRNzol Universal (Tiangen, Beijing, CN) and transcribed to cDNA with the FastKing One Step RT-qPCR kit (SYBR, Tiangen, Beijing, CN). The primers used for SYBR Green RT-qPCR were given in Table S1. Real-Time One-Step RT-qPCR was performed using a BioRad CFX96 Touch™ detection system (CA, USA). The cycling conditions were 50 °C for 30 min, and 95 °C for 3 min, followed by 40 cycles of 95 °C for 15 s, and 60 °C for 30 s. The

data of the gene expression were calculated using the $2^{-\Delta\Delta CT}$ method, and plotted as a gene heat map using GraphPad Prism 8.0.

Statistical analysis

All experiments were conducted independently in triplicate, and data are expressed as the mean ± SEM. Pairwise comparisons between multiple groups were performed using a one-way ANOVA with Tukey's multiple comparison test. Unpaired comparisons of numerical data between the two groups were performed using an unpaired *t*-test. Statistical analyses were performed using SPSS 22.0. Statistical values of $P < 0.05$ were regarded as statistically significant and represented by an asterisk (*). P values < 0.01 were regarded as more statistically significant and represented by double asterisks (**). P values < 0.001 were regarded as the most statistically significant and represented by triple asterisks (***).

Table S1. The primer sequences of mRNA.

Oligo	Species	Forward sequences 5'-3'	Reverse sequences 5'-3'	Product length (bp)
BATF3	Mouse	GGACGATGACAGGAAAGTTCG	ACCGAAGCTGCACAAAGTTC	248
IRF8	Mouse	TATGCCGCCTATGACACACA	TCCGGCCCATACAACTTAGG	155
ID2	Mouse	CCCAGAACAAGAAGGTGACC	TGATGTCCGTGTTCAGGGTG	171
NFIL3	Mouse	GCTTTGGACAGCGAGTTTGA	TTTGTGATGCCAGTGTTCCG	186
IL-12p40	Mouse	CATCATCAAACCAGACCCGC	TCTTTCTTGCGCTGGATTCG	150
CXCL9	Mouse	GAACGGAGATCAAACCTGCC	CGACGACTTTGGGGTGTTTT	159
CXCL10	Mouse	CCAAGTGCTGCCGTCATTTT	AATGATCTCAACACGTGGGC	183
IL-18	Mouse	GCTGTGACCCTCTCTGTGAA	TCCATCTTGTTGTGTCCTGGA	158
IL-15	Mouse	CTGAGGCTGGCATTCATGTC	TGCAACTGGGATGAAAGTCAC	171
IRF4	Mouse	TCCCCATTGAGCCAAGCATA	TCCTCTGTCCATTGTCGTCC	207
RELB	Mouse	TACAATGCTGGCTCCCTGAA	CGCTCTCCTTGTTGATTCGG	190

Oligo	Species	Forward sequences 5'-3'	Reverse sequences 5'-3'	Product length (bp)
IRF2	Mouse	GAGAGTGATGACCAGCCAGT	GGTACTGCTTGCCTTCGATG	166
NOTCH2	Mouse	AGTGTCGAGGTGGTCAAGAG	AGGGGTGAGAGGTGGAGTAT	246
RBP-J	Mouse	AGAGTGTGGTTTGGGGATGT	GAAGGTAAGGCTGGTGGAGT	180
STAT5a	Mouse	CAGAACACGTATGACCGCTG	TCTCCGTGTCCTGTGTGATC	196
STAT5b	Mouse	CGAAAGCAGCTGACGGATAC	CCATCGGTATCAAGGACGGA	184
CRLF2	Mouse	CTAGCCTCCTGTACCGCAAG	AGTTCCCGTGATGCTTCTCA	205
IL-7Rα	Mouse	AAAGTCCGATCCATTCCCCA	GGGAGACTAGGCCATACGAC	221
ZEB-2	Mouse	AGTGGCAGCAGTCCCTTTAT	TCCGTCTTGCAGTCCATCTT	215
KLF4	Mouse	AACTACCCTCCTTTCCTGCC	GTGTGGGTGGCTGTTCTTTT	152
CXCR5	Mouse	CCAGGAAAACGAAGCGGAAA	ATGACAATGTGGTAGGGCGA	222
TLR2	Mouse	CTGAGAATGATGTGGGCGTG	TTAAAGGGCGGGTCAGAGTT	161
TLR4	Mouse	AGGCAGCAGGTGGAATTGTA	GGTCCAAGTTGCCGTTTCTT	174

Supplementary figures

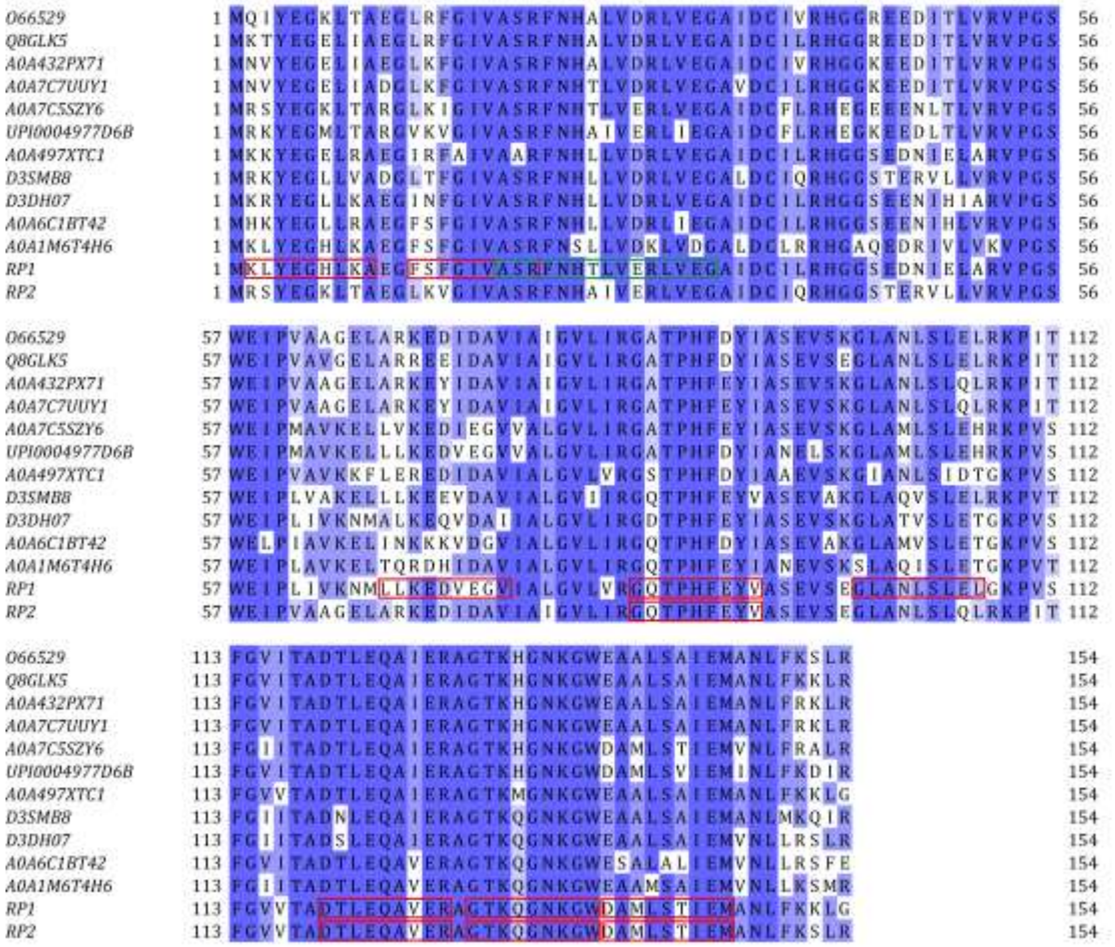

Figure S1. Sequence alignment of the homologous sequences, including the template AaLS subunit sequence (*UniProt Knowledgebase* accession number O66529). Similarity regions are shaded in blue. The sequence divergences highlight the evolutionary changes between AaLS and its related family members. Different colored rectangles represent the substituted epitopes. Red: MHCI molecule-binding epitope; green: MHCII molecule-binding epitope. All homologous sequences were similar to AaLS by more than 70%, indicating the reliability of homology modeling.

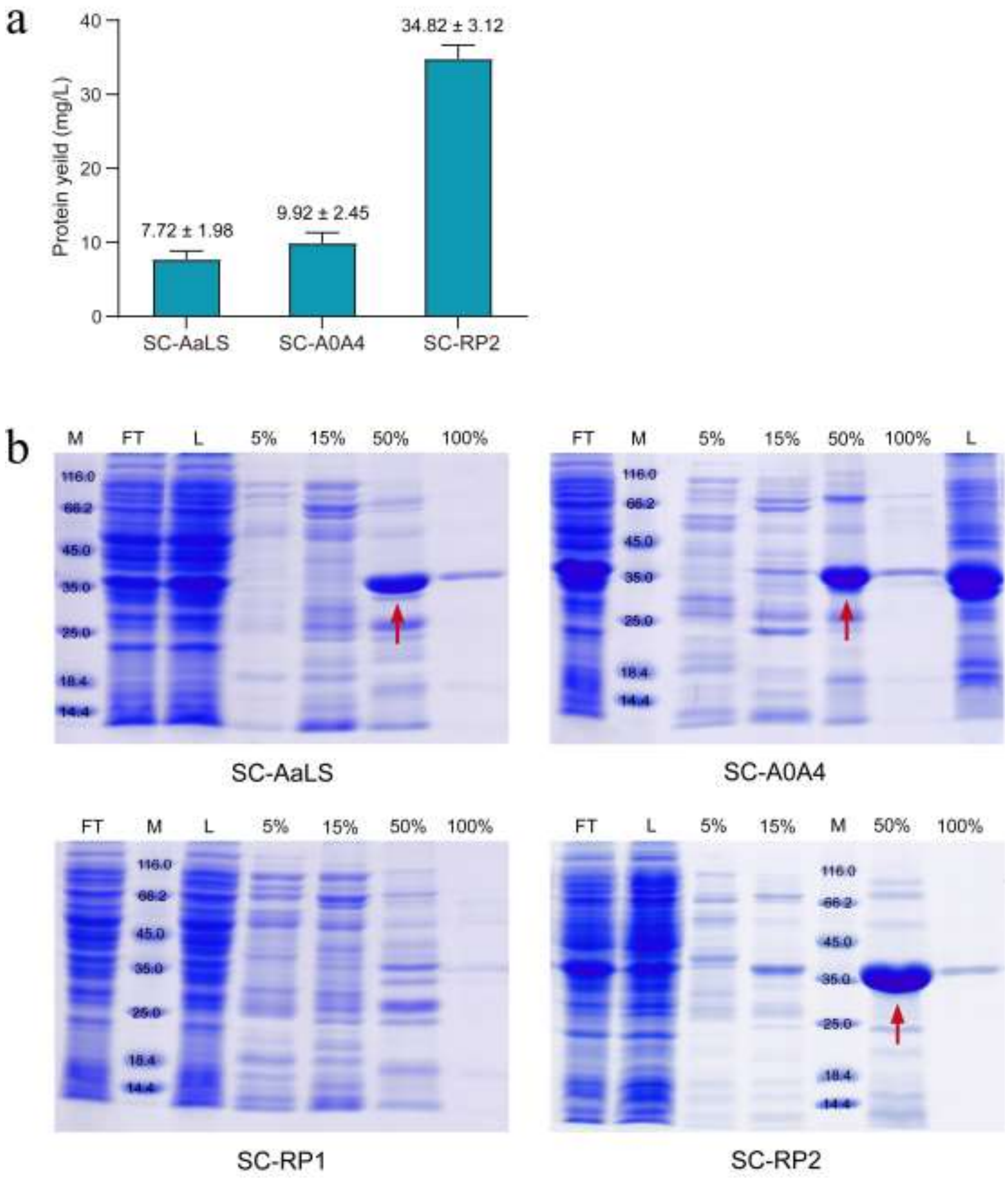

Figure S2. Protein expression and purification of the nanoscaffolds. a) Purified protein yields of SC-AaLS, SC-A0A4, and SC-RP$_T$ (n = 3). b) Electropherogram of proteins eluted with different concentration gradients of imidazole in an immobilized metal affinity chromatography (IMAC) assay. Soluble protein expression is labeled with a red arrow. FT: flow through, L: lysate, M: marker.

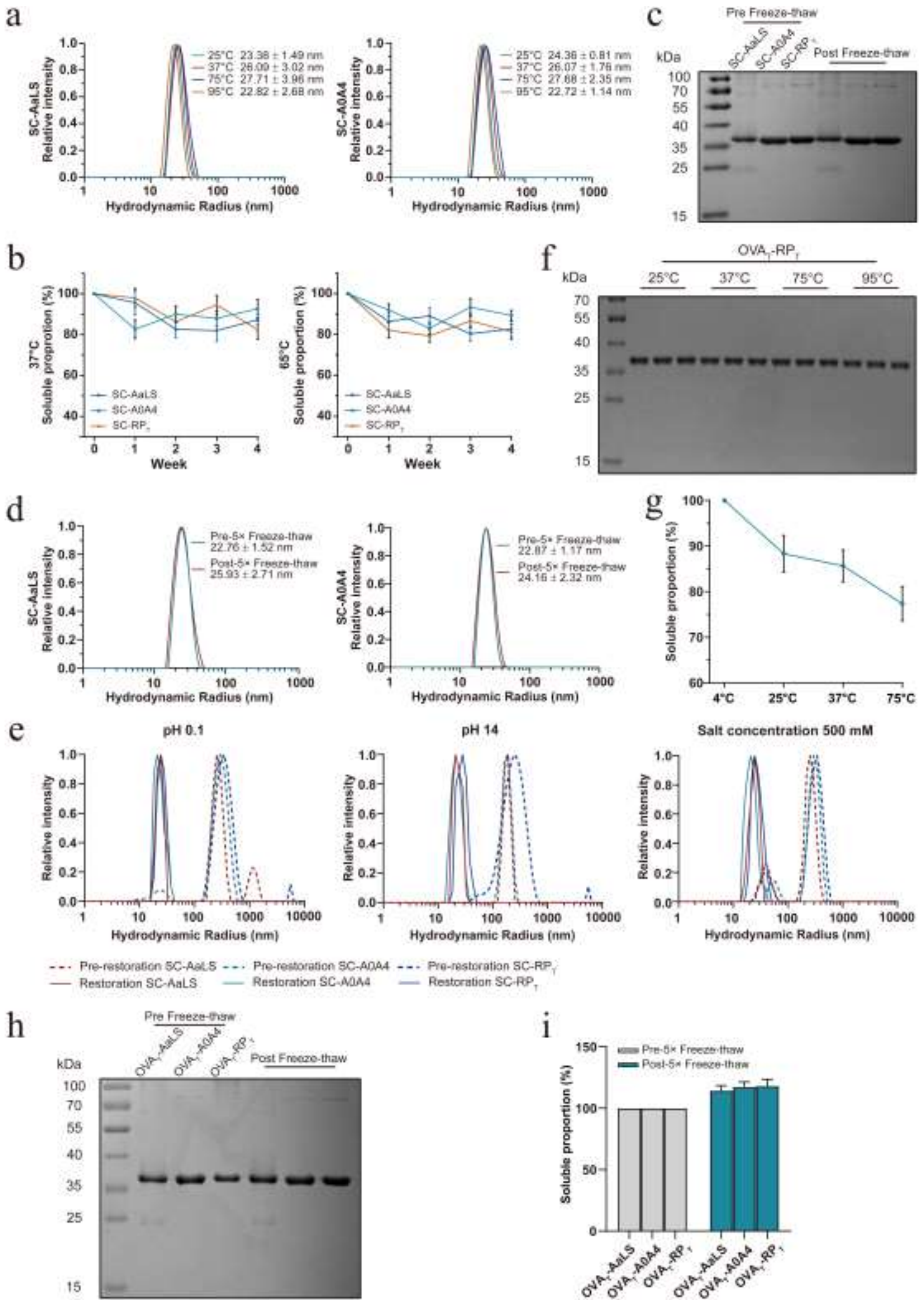

Figure S3. Stability analysis of the SC-RP$_T$ antigen delivery system. a) The integrity of SC-AaLS and SC-A0A4 after 2 h of incubation at different temperatures was determined using DLS (n = 3). b) Solubility curves of SC-AaLS, SC-A0A4, and SC-RP$_T$ at 37 °C or 65 °C for one month (n = 3).

c) The solubility of SC-AaLS, SC-A0A4, and SC-RP_T after 5× freeze-thawing was determined using SDS-PAGE with Coomassie staining. d) The integrity of SC-AaLS and SC-A0A4 after 5× freeze-thawing was determined using DLS (n = 3). e) The integrity of SC-AaLS, SC-A0A4, and SC-RP_T at pH 0.1 and 14 and a salt concentration of 500 mM was determined using DLS. f) The solubility and g) soluble proportion of OVA_T-RP_T after 48 h incubation at different temperatures were determined using SDS-PAGE with Coomassie staining (n = 3). h) The solubility and i) soluble proportion of OVA_T-AaLS, OVA_T-A0A4, and OVA_T-RP_T after 5× freeze-thawing were determined using SDS-PAGE with Coomassie staining (n = 3). Data are expressed as the mean ± SEM.

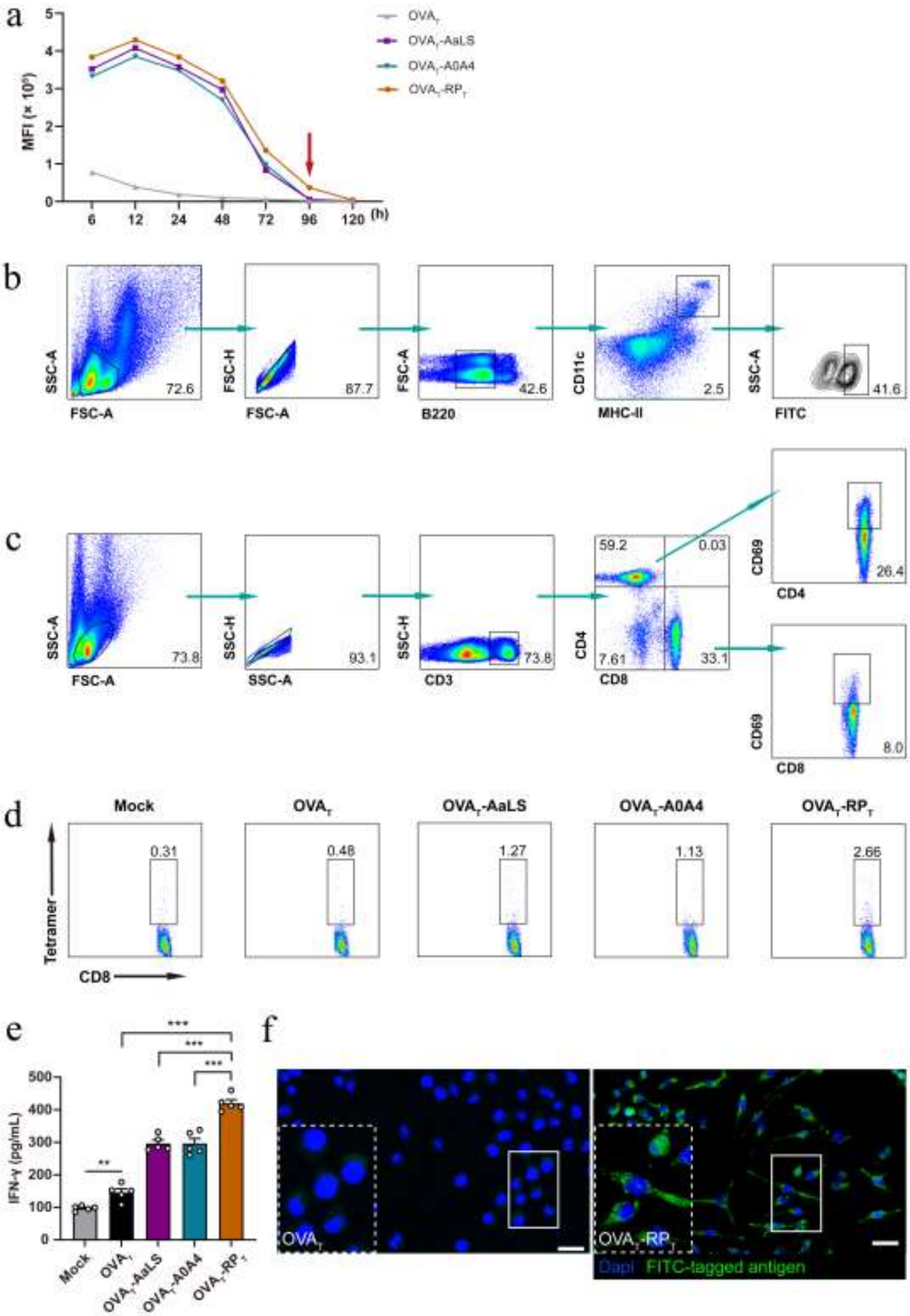

Figure S4. Antigen presentation and T cell immune response of SC-RP$_T$-derived tumor nanovaccines. a) Retention of free OVA$_T$, OVA$_T$-AaLS, OVA$_T$-A0A4, and OVA$_T$-RP$_T$ in inguinal lymph nodes at different time points (n = 3). The red arrow indicates that OVA$_T$-AaLS and

OVA_T-A0A4 were almost completely metabolized at 96 h post-injection, while OVA_T-RP_T still maintained partial fluorescence intensity. b) Gating strategy for FITC-labeled DCs. Inguinal lymph nodes were digested into single cells, and B220-non-B cells were further labeled as $CD11c^+$ $MHCII^+$ DCs. c) Gating strategy for activated $CD4^+$ and $CD8^+$ T cells. d) Representative flow cytometry plots of OVA_T-specific $CD8^+$ T cells in the mouse spleens. e) Splenocytes were stimulated *in vitro*, and the supernatant was collected to detect the secretion of IFN-γ using ELISA (n = 5). f) Immunofluorescence co-localization images of DC2.4 cells. Green staining indicates FITC-labeled antigen. Blue staining indicates DAPI-stained nuclei. Scale bar: 20 μm. Data are expressed as the mean ± SEM. **P* < 0.05, ***P* < 0.01, ****P*< 0.001; ns represents not significant.

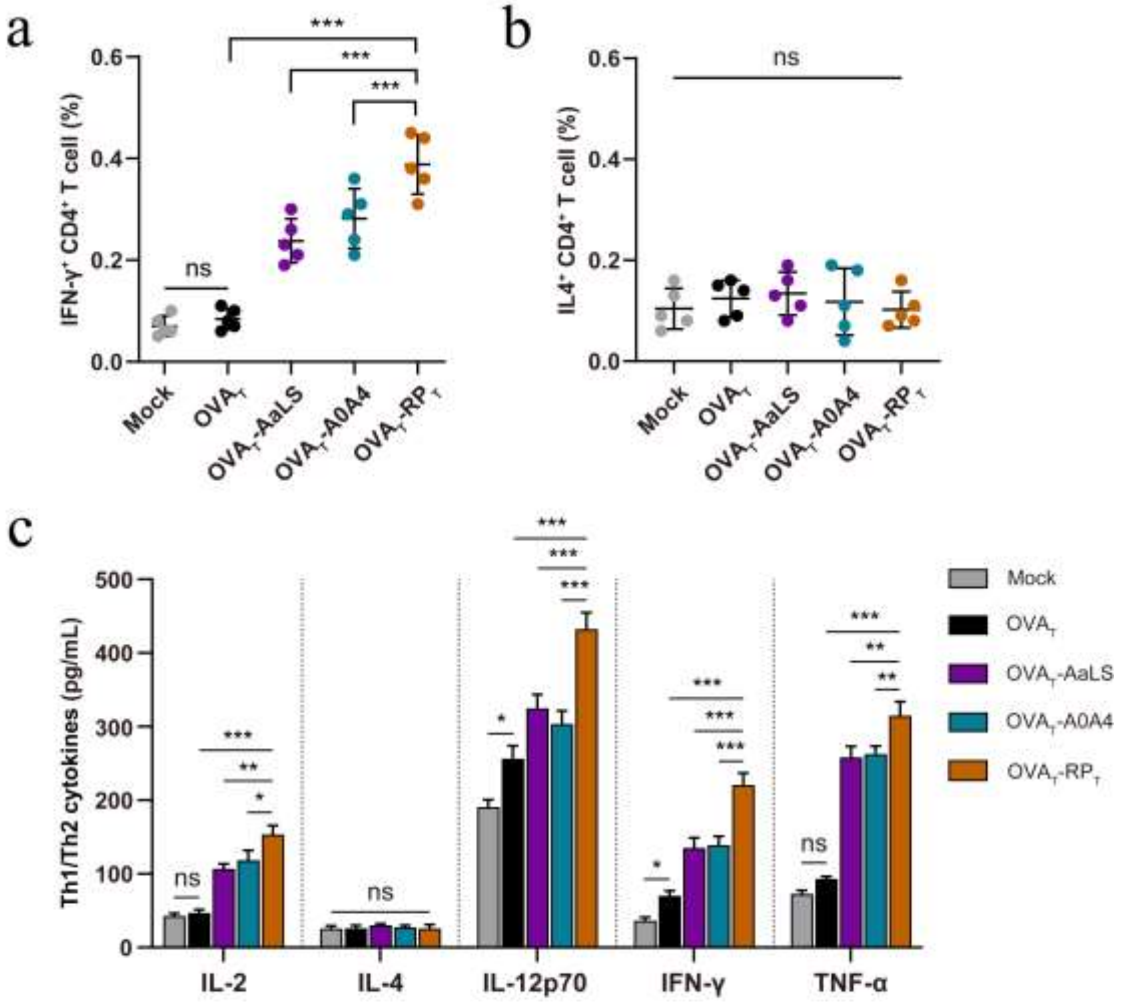

Figure S5. Th1/Th2 responses of SC-RP_T-derived tumor nanovaccine. a) Quantification of the percentage of IFN-γ^+ $CD4^+$ and b) IL-4^+ $CD4^+$ T cells in the spleen of immunized mice at 7 days post-administration (n = 5). c) The levels of Th1/Th2 cytokines in the peripheral blood of immunized mice at 7 days post-administration (n = 5). Data are expressed as the mean ± SEM. *P < 0.05, **P < 0.01, ***P < 0.001; ns represents not significant.

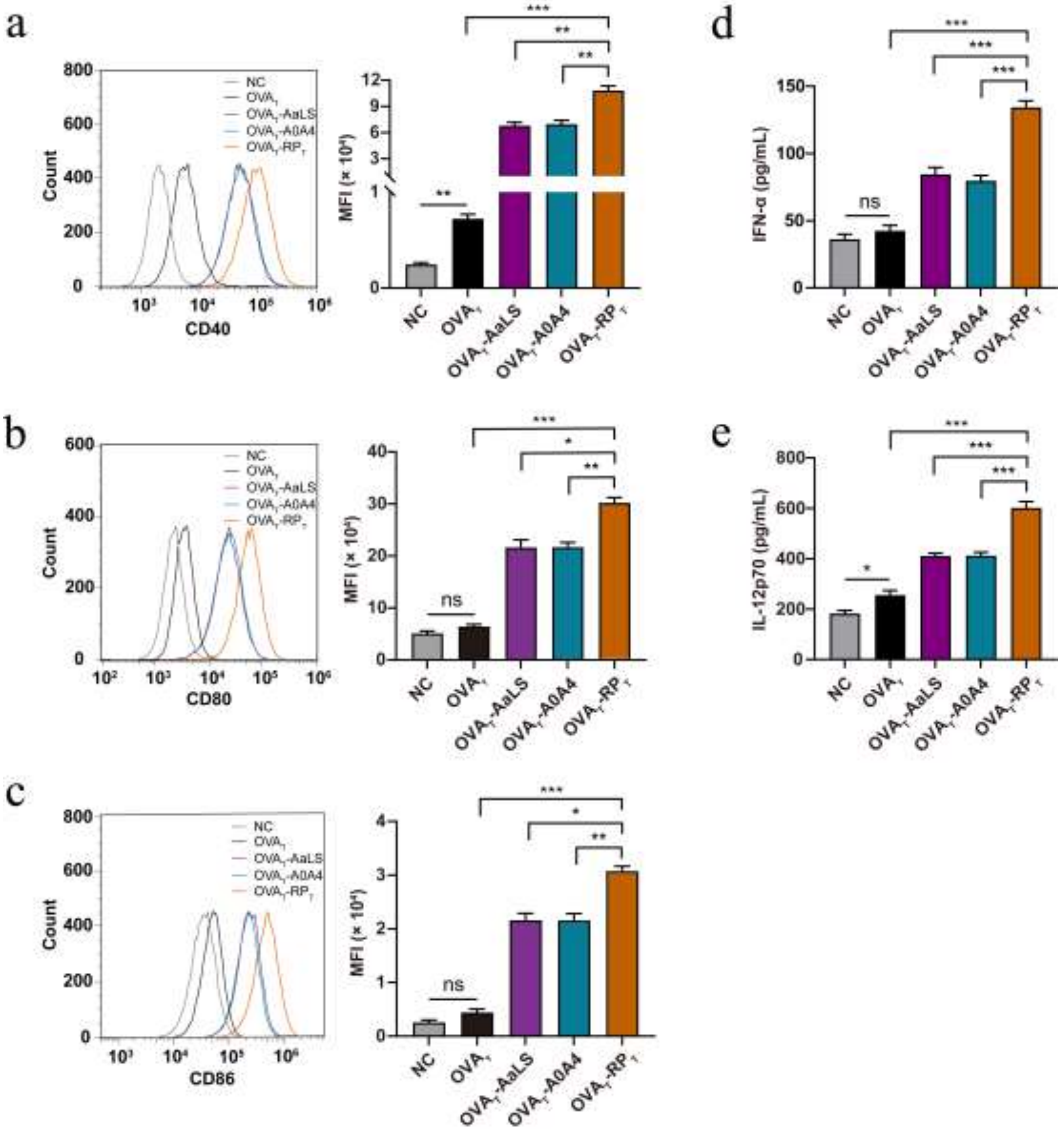

Figure S6. Signal detection for mature DCs. a) Representative flow cytometry plots and quantification of CD40, b) CD80 and c) CD86 in DC2.4 cells incubated with different antigens for

24 h (n = 3). d) The levels of IFN-α and e) IL-12p70 in the culture supernatants of DC2.4 cells incubated with different antigens for 24 h (n = 3). NC: negative control. Data are expressed as the mean ± SEM. *P < 0.05, **P < 0.01, ***P < 0.001; ns represents not significant.

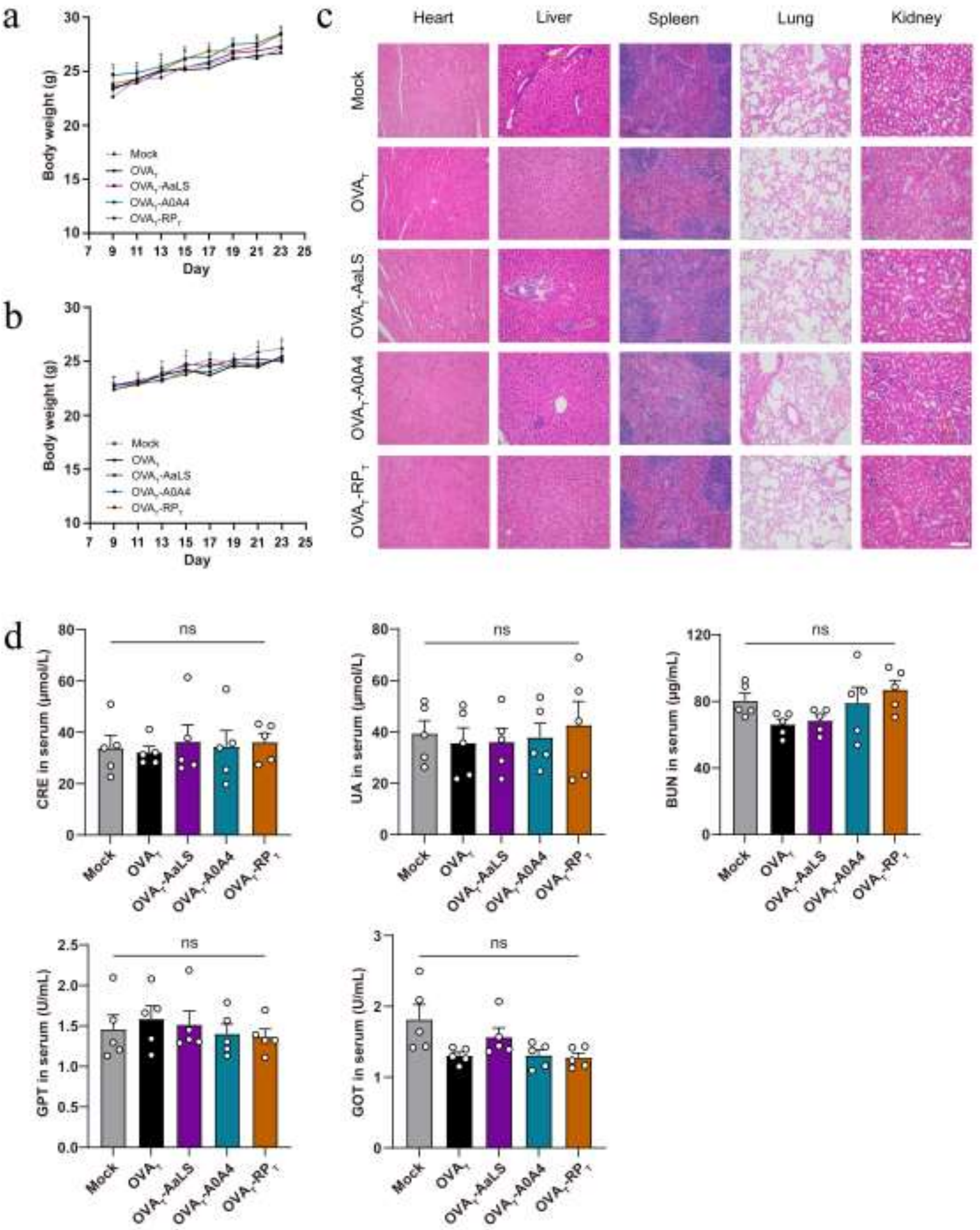

Figure S7. Safety analysis of SC-RP_T-derived tumor nanovaccines. a) Curves of body weight changes in prophylactic and b) therapeutic models as measured every other day until day 23 (n = 5). c) H&E staining of major organs in the prophylactic mouse models. Scale bar: 100 μm. d) Quantitative analysis of indicators for liver and renal function. CRE, UA, BUN, GPT, and GOT levels were measured in the prophylactic models (n = 5). Data are expressed as the mean ± SEM. ns represents not significant.

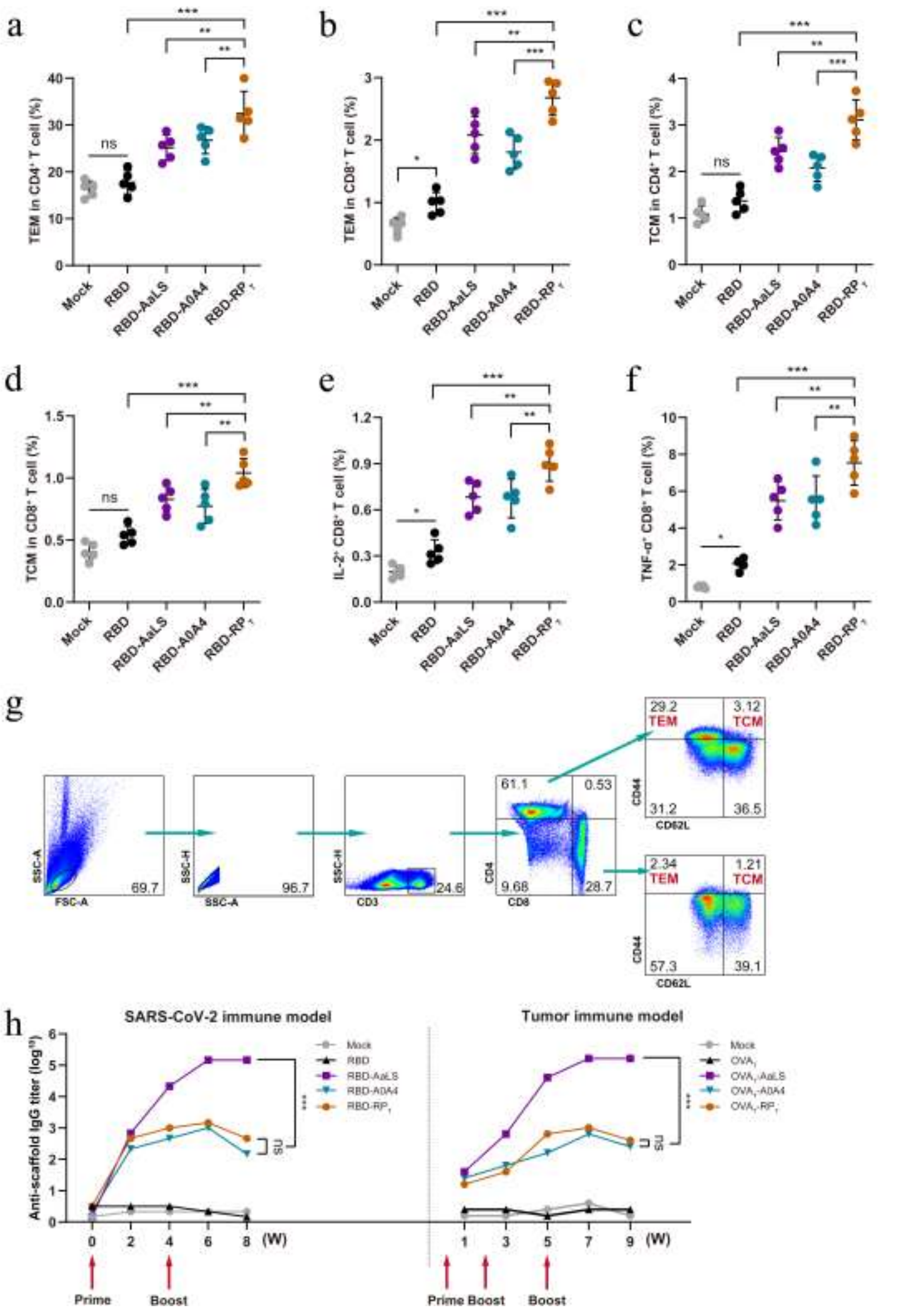

Figure S8. T cell immune response and anti-scaffold antibody response of SC-RP_T-derived SARS-CoV-2 nanovaccines. a) Quantification of the percentage of $CD4^+$ TEM, b) $CD8^+$ TEM, c) $CD4^+$ TCM, and d) $CD8^+$ TCM in the spleens of mice 8 weeks post-immunization (n = 5). e)

Quantification of the percentage of IL-2$^+$ CD8$^+$ and f) TNF-α$^+$ CD8$^+$ T cells in the spleens of mice 8 weeks post-immunization (n = 5). g) Gating strategy for TEM and TCM. h) Scaffold-specific IgG titers were calculated and plotted as a time-course curve in the SARS-CoV-2 (n = 6) and tumor (n = 5) immune models. Data are expressed as the mean ± SEM. *P < 0.05, **P < 0.01, ***P < 0.001; ns represents not significant.

Supplementary references

[1] Bruun TUJ, Andersson AC, Draper SJ, et al. Engineering a Rugged Nanoscaffold To Enhance Plug-and-Display Vaccination. ACS Nano. 2018, 12(9): 8855-8866.

[2] Lutz MB, Kukutsch N, Ogilvie AL, et al. An advanced culture method for generating large quantities of highly pure dendritic cells from mouse bone marrow. J Immunol Methods. 1999, 223(1): 77-92.
